# Prevalence of radiographic knee osteoarthritis in China: a national survey of thirty thousand, four hundred and fifty five individuals cross-sectional study

**DOI:** 10.1007/s00264-025-06643-9

**Published:** 2025-09-03

**Authors:** Hongzhi Lv, Zhiyong Hou, Yanbin Zhu, Ran Sun, Jianzhao Wang, Guangzhao Hou, Bing Yin, Song Liu, Guang Yang, Xiao Chen, Bo Liu, Peizhi Yuwen, Fei Zhang, Shilun Li, Juan Wang, Wei Chen, Yingze Zhang

**Affiliations:** The Third Hospital of Hebei Medical University, Shijiazhuang, China

**Keywords:** Knee osteoarthritis, National prevalence, Risk factors, Prediction model

## Abstract

**Background:**

Knee osteoarthritis (KOA) is a major health burden for adults and the elderly globally; however, national radiological and epidemiological data and predictive models for KOA are lacking in China. Most of the existing studies are limited to regional samples, which cannot accurately reflect disease burden and risk factors.

**Objectives:**

This study aimed to systematically analyze the prevalence and risk factors of adult radiology KOA in China for the first time using a national representative sample and to develop a prediction model to provide a basis for public health strategies.

**Methods:**

A multi-stage stratified random sampling method was employed to select 30 cities and 10 rural counties from 10 provinces, consisting of 30,455 participants aged 50 years and older who had lived in the area for at least five years. Knee X-rays were assessed using the Kellgren–Lawrence grading system, and demographic, clinical, and geographic data were collected. Samples were randomly divided into modeling and validation groups. A predictive model was developed using multiple logistic regression, and its performance was validated using receiver operating characteristic curves, calibration plots, and decision curve analysis. Further, an interactive web calculator based on R Shiny was developed.

**Results:**

This study enrolled 31,206 individuals. Questionnaires from 751 (2.5%) individuals were ultimately excluded due to missing items, insufficient responses, or logical errors. After exclusions, 30,455 (97.5%) individuals participated in the Chinese National KOA Study, consisting of 11,605 (38%) and 18,850 (62%) from urban and rural areas and 13,444 (44%) and 17,011 (56%) men and women, respectively. A total of 9,145 participants were diagnosed with radiographic KOA, and 3,515 participants, including 969 men and 2,546 women, had symptomatic knees. The population-weighted prevalence of radiographic KOA in China was 27.9 (95% confidence interval: 24.8–31.1) per 1000 people. A predictive model for KOA was developed, and its validity was verified among male and female patients. Significant risk factors for men included age, education, body mass index (BMI), central obesity, and residence in the hills; whereas, for women, age, education, BMI, previous knee impairment, more than two childbirths, and hypertension were risk factors. Two interactive web calculators based on R shiny were developed to access the probability of KOA. The website address for male patients was https://kneeosteoarthritisnomogram.shinyapps.io/DynNomapp/, and for female patients was https://femalekneeosteoarthritisnomogram.shinyapps.io/DynNomapp/.

**Conclusions:**

Our results provide detailed information on knee joint incidence, distribution, and risk factors, which is considered the latest clinical evidence basis for national healthcare planning and prevention efforts in China and other regions. To facilitate KOA prevention, public health policies focusing on risk factors for KOA, such as maintaining a healthy weight, implementing health management, and reducing underlying diseases, should be implemented. Further, men should avoid living in mountainous areas and women should have fewer childbirths and not have knee impairments.

**Supplementary Information:**

The online version contains supplementary material available at 10.1007/s00264-025-06643-9.

## Introduction

Knee osteoarthritis (KOA) is one of the most prevalent chronic diseases affecting the joints and their tissues, with a high health burden and impact not only on the patient but also on the healthcare system [1, 2]. Symptomatic activity-limited OA is estimated to affect 240 million people globally [3, 4]. Approximately 30% of people aged over 45 years have imaging evidence of KOA, and approximately half of them reported knee symptoms [5, 6]. The average cost of a patient with KOA is approximately $15,000 for direct medical expenses for KOA over their lifetime [7]. KOA is a significant drain on healthcare resources; however, epidemiological data on its prevalence across the country remains limited. Countries having no such data have to extrapolate from data from other regions, which is problematic because of the huge differences in prevalence. Until now, most epidemiological studies on KOA have assessed data from only one hospital or region [8]. With a population of more than 140 million, China has many ethnic groups, straddling the tropical temperate zone, with complex and diverse terrain, and coexistence of coastal and inland areas. Some studies in China have reported the epidemiology of KOA, but most were limited by small sample sizes, geographic areas, and lack of imaging data [9, 10]. In this research, we hypothesize that radiographic KOA prevalence in China exhibits distinct regional, age-, and gender-specific patterns; modifiable risk factors (e.g., body mass index [BMI], hypertension) and non-modifiable factors (e.g., geographic terrain) collectively impact KOA pathogenesis; a national predictive model integrating these factors can improve early disease identification.

The China Chronic Disease and Risk Factor Surveillance is an ongoing, regular, nationally representative cross-sectional study sponsored by the Chinese Center for Disease Control and Prevention that started in 2004 to monitor the prevalence of major noncommunicable diseases and their risk factors in China [11]. However, this survey did not collect KOA information. Several previous studies have identified risk factors for KOA, including obesity and overweight, comorbidity, occupational factors, physical activity, biomechanical factors, and dietary exposure [12]. However, the prevalence and risk factors of radiographic KOA in China remain unknown. Unlike previous single-center studies, we designed the Chinese National KOA Study (CNKS) to provide the first comprehensive and up-to-date national dataset of KOA prevalence rates, distribution, and risk factors throughout China. Based on the independent risk factors, a prediction model was developed to predict KOA and provide a reference for its clinical diagnosis and treatment, thereby bridging the evidence gap in low- and middle-income countries where KOA epidemiology is disproportionately understudied compared with high-income regions [45].

## Methods

### Sampling method and sample size

The sampling of the national survey was conducted in provinces, cities, counties, streets, towns, communities, and villages. At the provincial sampling level, 31 provinces, municipalities, or autonomous regions in mainland China were categorized into eastern, central, and western regions based on socioeconomic development and climate. Using stratified random sampling, ten provinces (autonomous regions or municipalities), comprising four from the east, two from the middle, and four from the west, were initially selected. Sampling was performed separately in urban and rural areas within each selected province (autonomous regions or municipalities).

All families in each neighborhood community and administrative village were interviewed, and all eligible members of each family aged 50 years and older living in their current residence for five years or more were invited to the survey. Total samples of 11,605 and 18,850 individuals were targeted in urban and rural areas, respectively.

### Participants and survey

The field survey was conducted from June 26, 2015, to March 19, 2016. Three survey teams were established and trained. Each team consisted of one radiologist, one joint surgeon, one radiographer, and six other trained members. Three physical examination hospital cars (Seeho Medical Company LTD, Guangzhou, China, each for one team) equipped with Siemens AXIOM Aristos Digital Radiograph were employed for the survey. Trained research team members personally interviewed all eligible household members aged 50 years and older. An alternative community or village was randomly selected in cases in which more than 10% of all families had moved or refused to participate. A standardized questionnaire was completed during the field survey. This questionnaire collected information about demographic characteristics, such as age, sex, occupation, education, Chinese ethnic nationality, marital status, body weight and height, waist circumference, geographical region, and residency category, whether born in famine years, chronic medical disease and medication history, lifestyle and dietary habit, history of disease and knee joint trauma, family history, and Per capita month income. The age at menopause and the number of children were recorded for women (Appendix I). The mean monthly temperature, rainfall, and relative humidity of the selected cities/counties in January and July over the past five years were extracted from meteorological observatories for further analysis.

KOA can be radiographically and clinically defined. The most predominant method for radiographic definition is the Kellgren–Lawrence (KL) radiographic grading system, which has been employed for more than 40 years. This overall joint scoring system grades KOA into five levels, ranging from 0 to 4, defining KOA by the presence of a definite osteophyte (grade 2), and more severe grades by the presumed successive appearance of joint space narrowing, sclerosis, cysts, and deformity.

All individuals were asked whether they experienced a symptomatic knee joint and underwent a physical examination of both knees. Symptoms and physical signs were recorded. All participants were instructed to take anteroposterior and lateral radiographs of the bilateral knees in physical examination hospital centres. The radiographs were uploaded into picture-archiving and communication systems. A radiologist and an orthopaedic surgeon with rich experience in KOA diagnosis and treatment identified whether the radiographic features of KOA existed in the X-ray films. If radiological KOA was confirmed, its severity was assessed using a KL grade scale, ranging from 0 to 4. The anatomical axes of the femur and tibia correspond to the diaphyseal midline of these long bones. The anatomical axes of the femoral and tibial diaphysis form a laterally opened angle named the anatomical femorotibial angle (aFTA). The aFTA was measured three times in the anteroposterior radiographs of the bilateral knees of each participant, and the mean value was recorded for further analysis.

Three quality control teams were established, with 10% of all questionnaires collected in the field being sampled by the quality control team to check for omissions and errors. This study was registered in the Chinese Clinical Trial Registry (number ChiCTR-EPC-15006133).

### Statistical analysis

All data were recorded in a written survey at each selected household and later entered into the EpiData version 3.1 software using the dual import program. Dually imported data were then compared, and the original printed versions were consulted and corrections were made accordingly in cases of mismatched information.

The counting data is presented as frequency (percentage), and normally distributed measurement data are expressed as mean ± standard deviation whereas those non-normally distributed were reported as the median (interquartile distance). The Proc Survey freq procedure in SAS version 9.2 was used to calculate the overall and sex-specific prevalence of symptomatic KOA following each risk factor strata. The prevalence trends between each tier were assessed by including the prevalence of each tier of these risk factors as a continuous variable in the univariate logistic regression model.

Moreover, R version 4.2.0 was used to assess the risk factors for KOA in women and men. We studied potential correlations between KOA and various factors of interest, including age, ethnic origin, education, occupation, cigarette smoking, alcohol drinking, calcium or vitamin D consumption, BMI, knee injury history, and urbanization, as well as menopause and number of children per woman. A separate design-based multiple logistic regression model was developed for each gender. In the modeling population, univariate analysis was conducted on general information, such as gender, ethnicity, occupation, education level, and meteorological factors, and variables with *P*-values of < 0.05 were screened out. The χ [2] test or Fisher exact probability method was used for comparison between counting data groups, and the test or Mann–Whitney U test was employed for comparison between measurement data groups. After Spearman’s rank correlation test, variables with *P*-values of < 0.05 and correlation coefficient r of > 0.5 could not be included in the model simultaneously. After removal, the remaining variables were included in the multivariate logistic regression for further analysis, and independent influencing factors associated with knee arthritis were obtained. A *P*-value of < 0.05 indicated significance. Based on the multifactor analysis, a nomogram was established to predict the risk of developing knee arthritis in each individual. Receiver operating characteristic (ROC) curves, calibration charts, and decision curve analysis (DCA) curves were used to assess the differentiation, calibration, and clinical effectiveness of the model through the overall evaluation, internal verification, and external verification.

## Results

Ten provinces (autonomous regions or municipalities) were initially selected employing stratified random sampling. Sampling was performed separately in urban and rural areas within each selected province (autonomous regions or municipalities). In the urban areas, 30 cities were selected (10 large, 10 mid-sized, and 10 small cities in the selected provinces and municipalities). Thirty-four neighborhood communities were sampled from 30 selected streets in these cities. In the rural areas, ten counties were sampled, and 37 administrative villages were selected from 20 chosen towns in these counties. All selected communities and villages accepted to participate during the sampling phase; however, one village contained fewer individuals than those recorded in the 2010 census data. Therefore, a single administrative village was replaced.

### Basic information of the CNKS model

This study selected 31,206 individuals. Questionnaires from 751 (2.5%) individuals were ultimately excluded due to missing items, insufficient responses, or logical errors. After exclusions, 30,455 (97.5%) individuals participated in the CNKS, consisting of 11,605 (38%) and 18,850 (62%) from urban and rural areas and 13,444 (44%) [64.1 ± 9.2 years old] and 17,011 (56%) [63.1 ± 9.0 years old] men and women, respectively. A total of 9,145 participants were diagnosed with radiographic KOA, among whom 7,900 had bilateral radiographic KOA, 576 had isolated right radiographic KOA, and 669 had isolated left radiographic KOA. Among the participants with radiographic KOA, 2,859 men and 6,286 women with an average age of 66.9 ± 9.0 years old were significantly older than the 21,310 participants without radiographic KOA (62.1 ± 8.7 years old, *P* < 0.05). Among those with KOA, 3,515 participants, including 969 men and 2,546 women with an average age of 66.0 ± 8.5 years old, reported symptomatic knees. Table 1 summarizes the radiographic KOA results that were assessed according to KL grade.

The average age of all participants was 61.8 ± 9.0 years. Among patients with radiographic KOA, mean ages were 65.9 ± 9.1 and 64.74 ± 8.91 years, BMIs were 24.1 ± 3.2 kg/m² and 24.5 ± 3.6 kg/m², and mean waist circumferences were 85.3 ± 8.5 and 83.3 ± 8.5 for males and females, respectively. The age of menopause was 50.2 ± 2.1 years old and the average number of children was 2.7 ± 1.3 for women. Of the male and female participants, 97.6% and 97.6% were of Han Nationality, 66.3% and 63.9% were from rural areas, 31.2% and 1.2% smoked before regular knee pain and morning stiffness onset, 28.3% and 0.8% regularly drank water before knee pain and morning stiffness presentation, 27.5% and 30.2% reported hypertension, 5.5% and 7.7% had diabetes, 22.1% and 25.4% regularly take anti-hypertensive drugs, 4.7% and 6.6% regularly take hypoglycemic drugs or insulin, 2.0% and 2.7% regularly take medications to treat digestive system diseases, and 1.5% and 2.4% regularly take vitamin D, calcium supplements, or antiosteoporosis drugs, respectively (Table 2).

The population-weighted prevalence of radiographic KOA in China was 27.9 (95% confidence interval [CI]: 24.8–31.1) per 1000 population, with a prevalence of 20.0 (95% CI: 17.1–23.0) per 1,000 population in males, which was significantly lower than that of 35.8 (95% CI: 32.2–39.5) per 1,000 population in females (Table 3). The population-weighted prevalence of radiographic KOA by individual characteristics and regions demonstrated no significant difference in prevalence among intergroups of ethnicity, menopause age, and long-term use of vitamin D or calcium, nor was there any significant difference in terms of geographical landform, urbanization, coastal, or innerland (Table 3). Among men and women, the highest prevalence rates per 1000 population were reported in the age of ≥ 80 years at 41.0 (31.6–50.4) and 65.1 (58.6–71.7), residing in the east region at 24.2 (20.4, 28.1) and 40.9 (36.0, 45.7), illiterate at 34.0 (24.3, 43.6) and 47.4 (39.1, 55.6), farmers at 23.2 (19.9, 26.4) and 39.1 (34.3, 44.0), BMI of > 30 kg/m2 at 29.0 (22.2, 35.8) and 48.9 (43.6, 54.3), per capita month income of < 1000 RMB/month at 23.7 (19.3, 28.1) and 38.3 (32.6, 44.1), nonsmoker at 22.2 (18.9, 25.6) and 36.1 (32.4, 39.7), and abstainer from alcohol drinking at 21.8 (18.7, 24.8) and 36.0 (32.4, 39.5), respectively. Among women, more than three children had the highest prevalence rate (48.2, 43.3–53.2). Individuals with a previous knee injury (40.8, 30.8–50.8), hypertension (36.8, 32.7–41.0), diabetes (33.3, 28.4–38.1), long-term use of anti-hypertensive drugs (38.6, 34.4–42.8), long-term use of anti-diabetic drugs (36.2, 30.9–41.6), long-term use of digestive drugs (33.5, 28.9–38.1), born in famine years (29.5, 26.2–32.7), and had central obesity based on waist circumference (34.8, 30.7–39.0) demonstrated a higher prevalence rate. Supplement Table 1 summarizes the population-weighted proportion rates of each category of meteorological factors, including temperature, rain, and humidity. The mean monthly amount of relative humidity in January of Q1 exhibited the highest prevalence rate at 25.6 (19.8, 31.4) and 43.3 (36.8, 49.8) in men and women, respectively.

### Clinical prediction model of KOA

This study included 13,444 male patients aged 64.1 ± 9.2 years, consisting of 11,753 and 1,691 in the modeling and external validation groups, respectively. A total of 2,859 (21.3%) patients were diagnosed with KOA. Compared with the non-KOA population in the modeling group, patients with KOA were predominantly people over 70 years of age, with high school or lower education, born in the year of famine, with a BMI of ≥ 24 kg/m^2^, centrally obese, suffering from hypertension, taking anti-hypertensive drugs, and residing in a rural area. Smoking and drinking and suffering from diabetes mellitus were less frequent, and the precipitation amount of January, the mean relative humidity of January, and the mean relative humidity of July were higher (*P* < 0.05, Supplement Table 2). Correlation analysis revealed correlation coefficients r of > 0.5 between hypertension and taking anti-hypertensive medication, January precipitation and January mean relative humidity and July mean relative humidity (*P* < 0.05). Hence, taking anti-hypertensive drugs, January mean relative humidity, and July mean relative humidity were excluded, and all remaining factors were included in the multivariate logistic regression analysis (Appendix II).

The analysis included 17,011 female patients, consisting of 15,034 in the modeling group and 1,977 in the external validation group, aged 63.1 ± 9.0 years, and 6,286 (37.0%) with KOA. Compared with non-KOA patients, a higher percentage of patients with KOA were aged 60 years or older, centrally obese, rural dwellers, residents in mountainous areas and basins, born in the year of famine, had educational attainment of elementary school or less, a BMI of ≥ 24 kg/m^2^, previous knee injury, ≥ three childbirths, hypertension, diabetes mellitus, long-term use of digestive medications, anti-hypertensive drugs, anti-glucose drugs, an average monthly income of less than 1,000 yuan, relative decreased smoking and drinking practices, and low average temperature in January, average precipitation in January and July, and average relative humidity in January and July (*P* < 0.05, Supplement Table 3). Correlation analysis revealed that central obesity, anti-hypertensive drug, anti-glucose drug, January mean temperature, and January and July mean relative humidity were highly correlated with other factors (*r* > 0.5, *P* < 0.05). These factors were excluded, and the remaining factors were included in the multivariate logistic regression analysis.

Multivariate analysis revealed that people aged 60 years and older (60–69 years, odds ratio [OR] = 1.762, 95% CI: 1.540–2.015; 70–79 years, OR = 2.627, 95% CI: 2.268–3.042; and ≥ 80 years, OR = 3.731, 95% CI: 3.068–4.538), those with junior high school education or less (illiterate, OR = 2.304, 95% CI: 1.688–3.143; elementary school, OR = 1.939, 95% CI: 1.447–2.599; junior high school, OR = 1.450, 95% CI: 1.081–1.947), BMI of ≥ 24 kg/m^2^ (24–27.9, OR = 1.581, 95% CI: 1.136–2.200; ≥28, OR = 1.924, 95% CI: 1.341–2.761), central obesity (OR = 1.170, 95% CI: 1.036–1.320), hypertension (OR = 1.228, 95% CI: 1.103–1.366), and residing in the hills (OR = 1.424, 95% CI: 1.206–1.681) were risk factors for developing KOA among men, with smoking (OR = 0.799, 95% CI: 0.717–0.891), alcohol consumption (OR = 0.877, 95% CI: 0.786–0.980), living in the city (OR = 0.814, 95% CI: 0.723–0.916) and in the highlands (OR = 0.683, 95% CI: 0.561–0.833), and average January precipitation (OR = 0.993, 95% CI 0.990–0.996) as their protective factors (Table 4).

**Fig. 1:**
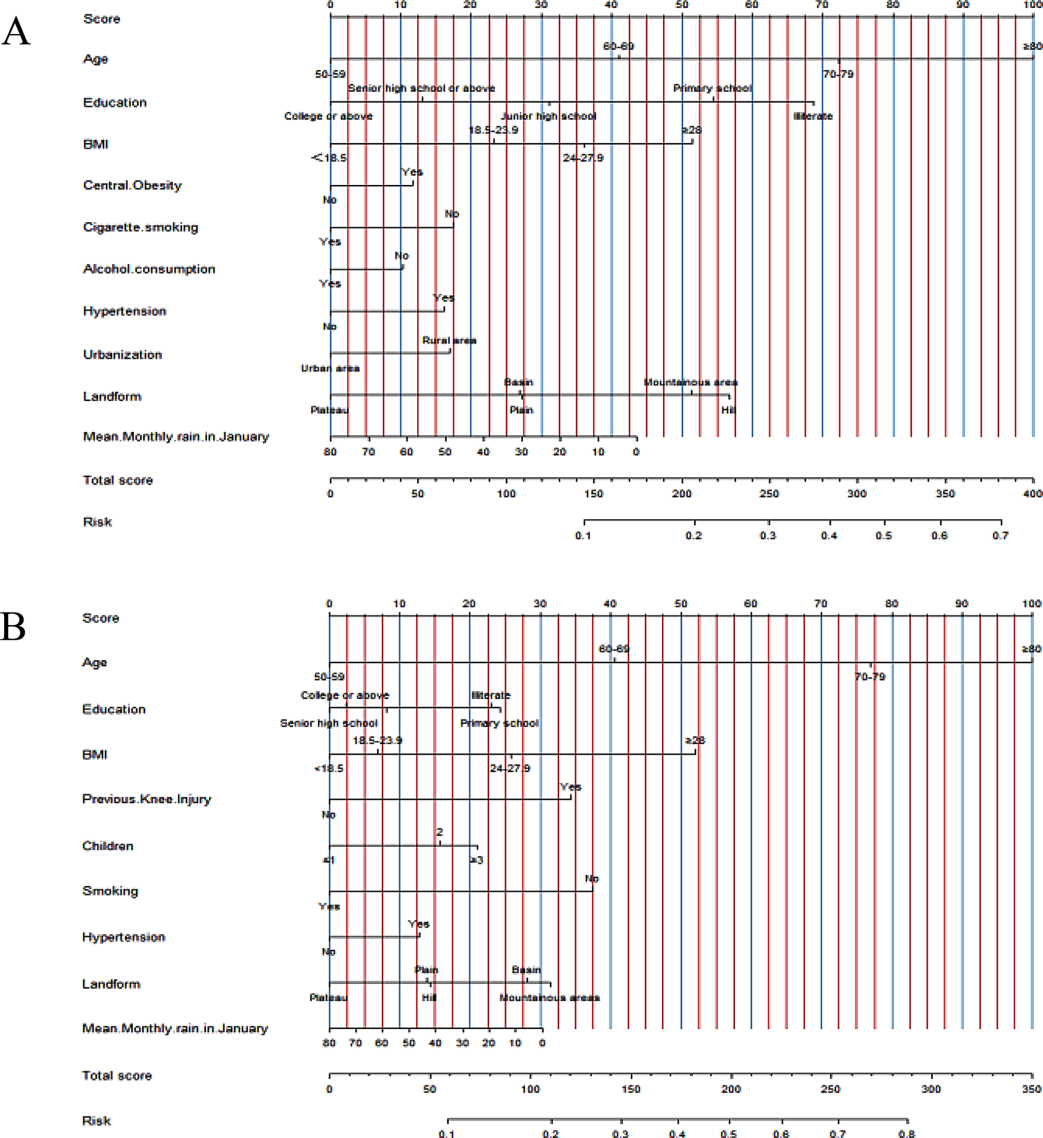
Nomograms of knee osteoarthritis in men (**A**) and women (**B**). *The score of each factor is read by the left scale, and the total score corresponds to the probability of disease risk at the bottom

Among female patients, age of 60 years and older (60–69 years, OR = 1.892, 95% CI: 1.720–2.080; 70–79 years, OR = 3.342, 95% CI: 2.968–3.764; ≥80 years, OR = 4.817, 95% CI: 4.030–5.757), an education level of junior high school and below (illiterate, OR = 1.415, 95% CI: 1.064–-1.880; elementary school, OR = 1.437, 95% CI: 1.090–1.894), BMI of ≥ 24 kg/m^2^ (24–27.9, OR = 1.495, 95% CI: 1.223–1.827; ≥28, OR = 2.257, 95% CI: 1.817–2.803), previous knee impairment (OR = 1.717, 95% CI: 1.200–2.457), ≥ 2 childbirths (2, OR = 1.282, 95% CI: 1.156–1.421; ≥3, OR = 1.392, 95% CI: 1.242–1.560), and hypertension (OR = 1.211, 95% CI: 1.115–1.316) were risk factors for developing KOA, with smoking (OR = 0.559, 95%: CI 0.414–0.756), residing in highland (OR = 0.811, 95% CI: 0.702–0.936), and average January precipitation (OR = 0.995, 95% CI: 0.992–0.997) as protective factors (Table 4). established based on the results of the multivariate logistic regression (Fig. 1).

**Table 1:** KL grade of radiographic and symptomatic KOA (${\rm\bar{x}}$±*s*)

K-L grade	Participants with radiographic KOA					Participants with symptomatic KOA			
	Men (*n*)	Age (year,${\rm\bar{x}}$±s)	Women (*n*)	Age (year,${\rm\bar{x}}$±s)		Men (*n*)	Age (year,${\rm\bar{x}}$±s)	Women (*n*)	Age (year,${\rm\bar{x}}$±s)
2 (most serious no more than grade 2)	1756	66.5 ± 9.1	3733	65.1 ± 8.9		186	70.6 ± 8.2	472	69.3 ± 8.0
3 (at least one knee)	700	68.3 ± 8.6	1646	67.2 ± 8.4		280	67.3 ± 8.4	782	66.0 ± 8.0
4 (at least one knee)	403	71.8 ± 8.3	907	70.7 ± 8.3		503	65.7 ± 8.5	1292	63.9 ± 8.4

*KL grade: Kellgren–Lawrence (KL) grading (0–4) assesses KOA severity radiographically. Grade 2: definite osteophytes; Grade 3: at least one knee with joint space nar­rowing, sclerosis, or cysts; Grade 4: severe joint space nar­rowing, sclerosis, cysts, and deformity

**Table 2:** Demographic characteristics of patients with KOA

issue	Men		Women		Total
	No	Yes	No	Yes	
Total	10,585	2859	10,725	6286	30,455
Age (year,${\rm\bar{x}}$±*s*)	61.4 ± 8.9	65.9 ± 9.1	59.5 ± 8.3	64.7 ± 8.9	61.8 ± 9.0
BMI (kg/m^2^,${\rm\bar{x}}$±*s*)	23.7 ± 2.9	24.1 ± 3.2	23.8 ± 3.2	24.5 ± 3.6	23.9 ± 3.2
waist circumference (cm,${\rm\bar{x}}$±*s*)	84.0 ± 7.8	85.3 ± 8.5	81.1 ± 7.0	83.3 ± 8.5	83.0 ± 7.9
Menopause age (years,${\rm\bar{x}}$±*s*)			50.2 ± 2.1	50.2 ± 2.1	50.2 ± 2.1
Number of children (*n*,${\rm\bar{x}}$±*s*)			2.3 ± 1.1	2.7 ± 1.3	2.4 ± 1.2
Ratio of current weight to weight at 20 years old (${\rm\bar{x}}$±*s*)	1.1 ± 0.1	1.1 ± 0.2	1.1 ± 0.2	1.2 ± 0.2	1.1 ± 0.2
The Proportion of Han Nationality among all participants (%)	97.6	97.6	97.6	97.6	97.6
The proportion of participants from rural areas among all participants (%)	60.9	66.3	60.6	63.9	61.9
The proportion of participants who smoke before the presentation of regular knee pain and morning stiffness (%)	39.6	31.2	1.9	1.15	17.6
The proportion of participants who regularly drink before the presentation of knee pain and morning stiffness (%)	34.8	28.3	1.2	0.8	15.4
The proportion of participants with hypertension among all participants (%)	20.3	27.5	21.1	30.2	23.3
The proportion of participants with diabetes among all participants (%)	5.7	5.5	5.6	7.7	6.0
The proportion of participants regularly taking antihypertensive drugs among all participants (%)	15.5	22.1	17.1	25.4	18.7
The proportion of participants regularly taking hypoglycemic drugs or insulin among all participants (%)	4.5	4.7	4.3	6.6	4.9
The proportion of all participants regularly taking medications to treat digestive system diseases(%)	2.1	2.0	1.9	2.7	2.2
The proportion of participants regularly taking Vitamin D, Calcium supplement or anti-osteoporosis drugs among all participants (%)	1.1	1.5	2.2	2.4	1.8

* BMI was categorized as <18.5, 18.5-23.9, 24-27.9, and ≥28 kg/m². Smoking/alcohol consumption refers to habits before the onset of regular knee pain or morning stiffness. Hypertension, diabetes, and medication use were based on self-report or clinical records. For women, menopause age and number of children were directly recorded

**Table 3:** Weighted prevalence of KOA and statistical test results in different population groups

Items	Sample size	Population-weighted prevalence (per 1,000) and 95% CI				Items	Sample size	Population-weighted prevalence (per 1,000) and 95% CI		
		Male	Female	Total				Male	Female	Total
**Total**	30, 455	20.0 (17.1, 23.0)	35.8 (32.2, 39.5)	27.9 (24.8, 31.1)		**Per capita month income**				
**Age (years)**						< 1000 RMB/month	3159	23.7 (19.3, 28.1)	38.3 (32.6, 44.1)	31.8 (27.4, 36.3)
50–59	10,228	12.7 (10.1, 15.2)	22.2 (19.5, 24.9)	17.3 (14.9, 19.8)		1000–1999 RMB/month	24,264	20.4 (17.4, 23.4)	36.0 (32.3, 39.8)	28.2 (25.0, 31.5)
60–69	12,310	\	38.9 (33.8, 44.0)	30.3 (26.4, 34.1)		> 2000 RMB/month	3032	13.6 (10.9, 16.3)	29.1 (24.6, 33.6)	19.9 (16.4, 23.4)
70–79	6211	31.9 (28.0, 35.8)	55.3 (50.9, 59.7)	43.9 (39.9, 47.8)		P-value for trend test		< 0.001	0.028	0.001
80+	1706	41.0 (31.6, 50.4)	65.1 (58.6, 71.7)	55.0 (47.8, 62.3)		**Smoking**				
P-value for trend test		< 0.001	< 0.001	< 0.001		Non-smoker	25,094	22.2 (18.9, 25.6)	36.1 (32.4, 39.7)	30.9 (27.5, 34.2)
**Ethnicity**						< 100k cigarettes	1128	15.7 (11.7, 19.8)	22.7 (14.2, 31.2)	16.3 (12.3, 20.3)
Han nationality	29,715	20.0 (17.1, 23.0)	36.0 (32.4, 39.6)	28.0 (24.9, 31.2)		100–299k cigarettes	3543	16.7 (13.3, 20.1)	28.6 (16.0, 41.2)	17.3 (13.8, 20.9)
Other nationalities	740	19.9 (12.0, 27.9)	25.6 (12.2, 39.0)	23.0 (12.8, 33.1)		> 300k cigarettes	690	17.4 (14.4, 20.4)	25.0 (10.8, 39.2)	17.5 (14.7, 20.4)
P-value for difference test		0.982	0.156	0.354		P-value for trend test		0.002	0.006	< 0.001
**Urbanization**						**Alcohol Drinking**				
Urban area	11,605	20.1 (15.8, 24.3)	36.1 (30.6, 41.6)	28.0 (23.2, 32.8)		Abstainer	25,778	21.8 (18.7, 24.8)	36.0 (32.4, 39.5)	30.4 (27.1, 33.6)
Rural area	18,850	20.0 (16.1, 23.9)	35.5 (31.2, 39.8)	27.9 (24.2, 31.6)		< 300 kg	2623	16.2 (12.0, 20.4)	26.0 (17.5, 34.5)	16.6 (12.7, 20.6)
P-value for difference test		0.987	0.865	0.973		300–599 kg	1121	17.9 (14.9, 21.0)	42.7 (19.5, 66.0)	18.3 (15.4, 21.2)
**Region**						> 600 kg	933	17.6 (14.1, 21.2)	22.1 (0.0, 55.0)	17.7 (13.8, 21.7)
East	15,081	24.2 (20.4, 28.1)	40.9 (36.0, 45.7)	32.6 (28.5, 36.7)		P-value for trend test		0.003	0.097	< 0.001
Central	8768	14.0 (12.6, 15.5)	28.8 (26.9, 30.7)	21.4 (19.7, 23.1)		**Menopause age**				
West	6606	16.9 (14.2, 19.7)	31.6 (27.3, 35.9)	24.2 (20.9, 27.5)		≤ 45 years	480	/	38.7 (31.6, 45.8)	38.7 (31.6, 45.8)
P-value for difference test		< 0.001	< 0.001	< 0.001		46–50 years	8962	/	36.7 (32.4, 41.1)	36.7 (32.4, 41.1)
**Landform**						> 50 years	7235	/	35.6 (32.3, 38.8)	35.6 (32.3, 38.8)
Plain	23,631	19.8 (16.0, 23.5)	36.3 (31.4, 41.2)	28.0 (23.8, 32.2)		P-value for trend test			0.981	0.981
Mountainous areas	3637	22.2 (16.5, 27.9)	35.8 (29.2, 42.4)	29.1 (23.5, 34.7)		**Number of Children**				
Plateau	2228	13.9 (9.6, 18.2)	29.4 (21.3, 37.5)	21.7 (15.8, 27.7)		≤ 1	3780	/	22.4 (19.9, 24.9)	22.4 (19.9, 24.9)
Basin	959	26.5 (19.6, 33.3)	48.4 (40.6, 56.1)	37.4 (30.3, 44.4)		2	6505	/	31.1 (26.3, 35.9)	31.1 (26.3, 35.9)
P-value for difference test		0.188	0.381	0.293		≥ 3	6726	/	48.2 (43.3, 53.2)	48.2 (43.3, 53.2)
**Coastal or Innerland**						P-value for trend test			< 0.001	< 0.001
Coastal	8899	21.5 (17.6, 25.5)	36.6 (32.0, 41.2)	29.1 (25.2, 32.9)		**Previous Knee Injury**				
Innerland	21,556	19.2 (15.3, 23.2)	35.4 (30.5, 40.4)	27.4 (23.0, 31.7)		No	30,198	20.0 (17.1, 22.9)	35.7 (32.0, 39.3)	27.9 (24.7, 31.0)
P-value for difference test		0.415	0.738	0.553		Yes	257	22.5 (12.8, 32.1)	52.1 (38.3, 66.0)	40.8 (30.8, 50.8)
**Education**						P-value for difference test		0.569	0.017	2.000
Illiterate	4046	34.0 (24.3, 43.6)	47.4 (39.1, 55.6)	42.5 (34.0, 51.1)		**Hypertension**				
Primary school	13,188	22.2 (17.8, 26.5)	38.8 (33.2, 44.5)	31.0 (26.0, 35.9)		No	23,360	18.5 (16.0, 21.0)	32.6 (29.4, 35.9)	25.4 (22.8, 28.0)
Junior high school	8997	17.2 (14.3, 20.1)	29.1 (25.1, 33.1)	22.5 (19.8, 25.2)		Yes	7095	26.1 (21.8, 30.4)	46.1 (41.7, 50.6)	36.8 (32.7, 41.0)
Senior high school	3288	14.3 (11.7, 16.9)	27.1 (20.9, 33.2)	20.1 (16.5, 23.6)		P-value for difference test		< 0.001	< 0.001	< 0.001
College or above	936	11.3 (6.1, 16.4)	23.1 (17.2, 29.0)	15.4 (11.4, 19.5)		**Diabetes**				
P-value for trend test		< 0.001	< 0.001	< 0.001		No	28,615	20.1 (17.2, 23.0)	35.2 (31.6, 38.8)	27.6 (24.5, 30.8)
**Occupation**						Yes	1840	19.3 (14.3, 24.3)	46.2 (40.4, 52.1)	33.3 (28.4, 38.1)
Officer	1330	13.9 (9.5, 18.3)	29.9 (21.6, 38.1)	20.2 (15.3, 25.2)		P-value for difference test		< 0.001	< 0.001	< 0.001
Office Clerk	674	14.8 (11.3, 18.4)	20.3 (16.0, 24.5)	16.8 (14.3, 19.2)		**Long time use of VD or calcium**				
Technician	933	20.3 (14.2, 26.3)	32.4 (25.9, 38.9)	26.4 (22.2, 30.7)		No	29,911	20.0 (17.1, 23.0)	35.7 (32.0, 39.4)	27.8 (24.7, 31.0)
Business and service personnel	2739	20.3 (12.7, 27.8)	29.0 (24.0, 34.1)	25.5 (19.7, 31.4)		Yes	544	20.9 (10.5, 31.4)	39.6 (32.8, 46.4)	33.3 (27.2, 39.4)
Farmer	15,717	23.2 (19.9, 26.4)	39.1 (34.3, 44.0)	31.5 (27.7, 35.3)		P-value for difference test		0.863	0.289	0.072
Manual worker and transportation personnel	6055	14.7 (12.0, 17.4)	30.7 (27.7, 33.8)	20.6 (17.7, 23.4)		**Long time use of anti-hypertensive drug**				
Other	1730	19.8 (14.2, 25.4)	34.5 (27.8, 41.3)	28.6 (22.5, 34.7)		No	24,748	18.7 (16.2, 21.3)	32.9 (29.7, 36.2)	25.7 (23.0, 28.3)
Unemployed	1277	17.7 (12.5, 22.8)	33.6 (27.3, 39.8)	28.3 (22.9, 33.8)		Yes	5707	27.1 (22.4, 31.8)	47.6 (43.0, 52.2)	38.6 (34.4, 42.8)
P-value for difference test		< 0.001	< 0.001	< 0.001		P-value for difference test		< 0.001	< 0.001	< 0.001
**BMI**						**Long time use of anti-diabetic drugs**				
< 18.5	917	15.1 (9.9, 20.2)	39.5 (32.9, 46.2)	30.7 (25.4, 36.0)		No	28,968	20.0 (17.1, 22.9)	35.2 (31.6, 38.8)	27.6 (24.5, 30.7)
18.5–23.9	15,692	19.1 (16.5, 21.7)	32.2 (28.8, 35.7)	25.3 (22.6, 28.1)		Yes	1487	20.8 (15.7, 26.0)	50.0 (43.6, 56.4)	36.2 (30.9, 41.6)
24-27.9	10,675	21.7 (17.9, 25.4)	40.5 (37.1, 43.9)	31.9 (28.4, 35.3)		P-value for difference test		0.697	< 0.001	< 0.001
≥ 28	3171	29.0 (22.2, 35.8)	48.9 (43.6, 54.3)	40.6 (35.1, 46.2)		**Long time use of treating digestive drug**				
P-value for trend test		< 0.001	< 0.001	< 0.001		No	29,633	20.0 (17.1, 23.0)	35.7 (32.0, 39.4)	27.9 (24.7, 31.0)
**Central obesity based on waist circumference**						Yes	822	20.3 (13.8, 26.7)	44.8 (38.6, 51.0)	33.5 (28.9, 38.1)
No	15,284	18.7 (16.2, 21.3)	32.2 (29.4, 34.9)	25.4 (23.0, 27.8)		P-value for difference test		0.938	0.018	0.037
Yes	15,171	23.7 (19.9, 27.6)	45.3 (40.9, 49.8)	34.8 (30.7, 39.0)						
P-value for difference test		< 0.001	< 0.001	< 0.001						
**Born in Famine years**										
No	28,277	21.0 (18.0, 24.1)	37.8 (34.0, 41.6)	29.5 (26.2, 32.7)						
Yes	2178	12.3 (9.1, 15.4)	20.5 (16.8, 24.2)	16.4 (13.5, 19.2)						
P-value for difference test		< 0.001	< 0.001	< 0.001						

*The trend test was performed using the Cochran-Armitage test to assess the trend of prevalence changes with stratifying factors.The inter-group difference test was performed using the χ² test to compare the statistical significance of differences in prevalence between subgroups

population-weighted prevalence: prevalence adjusted for demographic factors; 95% CI: 95% confidence interval.

**Table 4:** Multivariate logistic regression analysis of the model population

Risk factors	OR (95%CI)	
	Males	Females
**Age(years)**		
50–59	Reference	Reference
60–69	1.762(1.540,2.015)	1.892(1.720,2.080)
70–79	2.627(2.268,3.042)	3.342(2.968,3.764)
≥ 80	3.731(3.068,4.538)	4.817(4.030,5.757)
**Education**		
College or above	Reference	Reference
Illiterate	2.304(1.688,3.143)	1.415(1.064,1.880)
Primary school	1.939(1.447,2.599)	1.437(1.090,1.894)
Junior high school	1.450(1.081,1.947)	1.118(0.849,1.473)
Senior high school	1.156(0.841,1.591)	0.973(0.728,1.300)
**Born in famine year**		
Yes	Reference	Reference
No	0.827(0.655,1.044)	0.976(0.829,1.149)
**BMI(kg/m2)**		
< 18.5	Reference	Reference
18.5–23.9	1.342(0.972,1.853)	1.113(0.914,1.355)
24-27.9	1.581(1.136,2.200)	1.495(1.223,1.827)
≥ 28	1.924(1.341,2.761)	2.257(1.817,2.803)
**Central Obesity**		
No	Reference	Reference
Yes	1.170(1.036,1.320)	
**Previous Knee Injury**		
No	Reference	Reference
Yes		1.717(1.200,2.457)
**Children**		
≤ 1	Reference	Reference
2		1.282(1.156,1.421)
≥ 3		1.392(1.242,1.560)
**Smoking**		
No	Reference	Reference
Yes	0.799(0.717,0.891)	0.559(0.414,0.756)
**Alcohol consumption**		
No	Reference	Reference
Yes	0.877(0.786,0.980)	0.971(0.682,1.383)
**Hypertension**		
No	Reference	Reference
Yes	1.228(1.103,1.366)	1.211(1.115,1.316)
**Diabetes**		
No	Reference	Reference
Yes		1.102(0.957,1.268)
**Digestive drug**		
No	Reference	Reference
Yes		1.077(0.863,1.344)
**Urbanization**		
No	Reference	Reference
Yes	0.814(0.723,0.916)	0.951(0.869,1.042)
**Landform**		
Plain	Reference	Reference
Mountainous area	1.384(0.783,2.445)	1.358(0.855,2.157)
Plateau	0.683(0.561,0.833)	0.811(0.702,0.936)
Hill	1.424(1.206,1.681)	1.037(0.909,1.182)
Basin	1.015(0.804,1.282)	1.210(0.973,1.504)
**Mean Monthly income (RMB/month)**		
> 2000	Reference	Reference
< 1000	1.237(0.999,1.533)	0.895(0.755,1.061)
1000–1999	1.080(0.917,1.271)	0.913(0.797,1.045)
**Mean Monthly rain in January (mm)**	0.993(0.990,0.996)	0.995(0.992,0.997)
**Mean Monthly rain in July(mm)**		1.000(0.999,1.001)

**Fig. 2:**
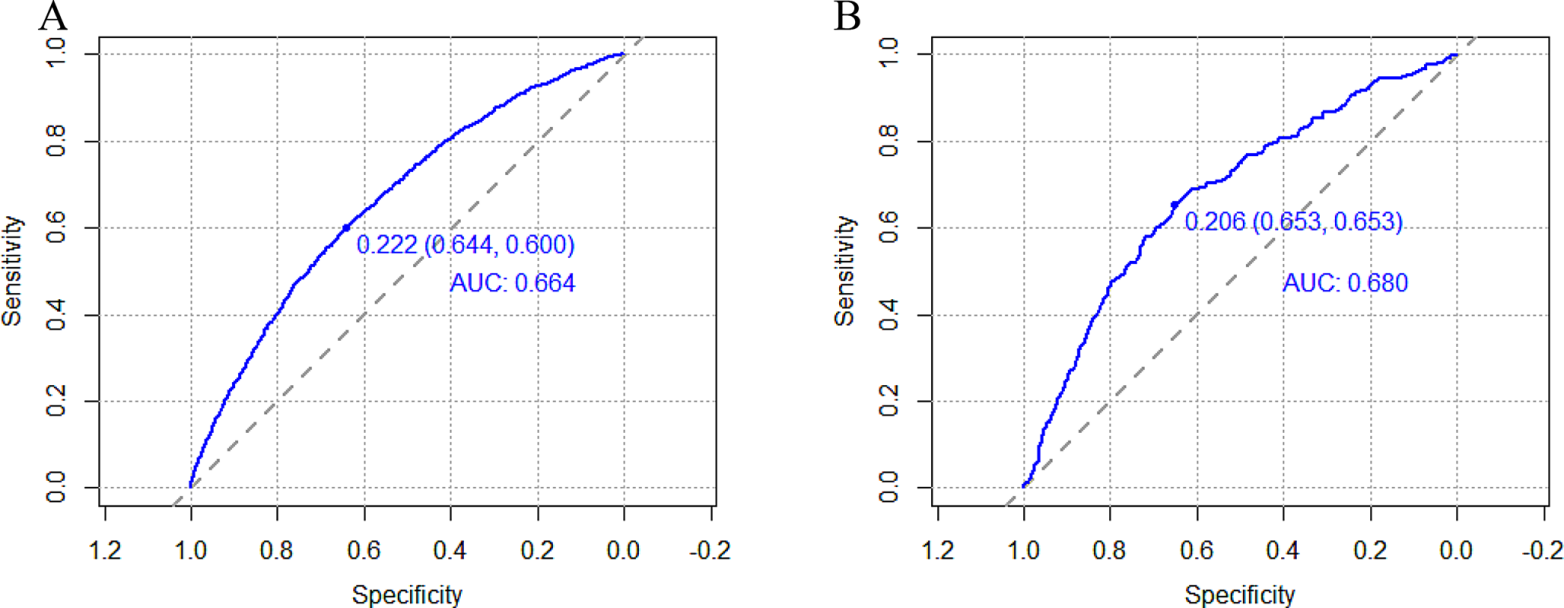
ROC curves for the prediction models of knee osteoarthritis in the male modeling group (**A**) and external validation group (**B**)

**Fig. 3:**
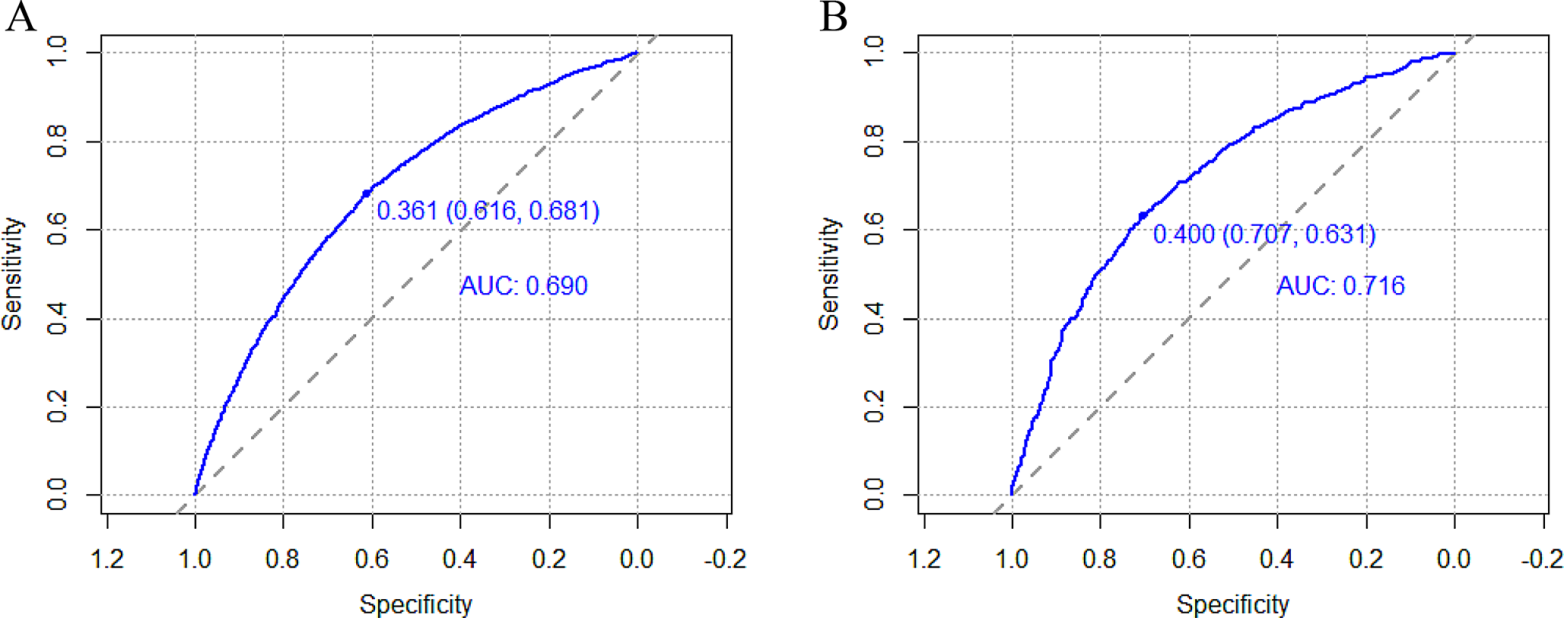
ROC curves for the prediction models of knee osteoarthritis in the female modeling group (**A**) and external validation group (**B**)

**Fig. 4:**
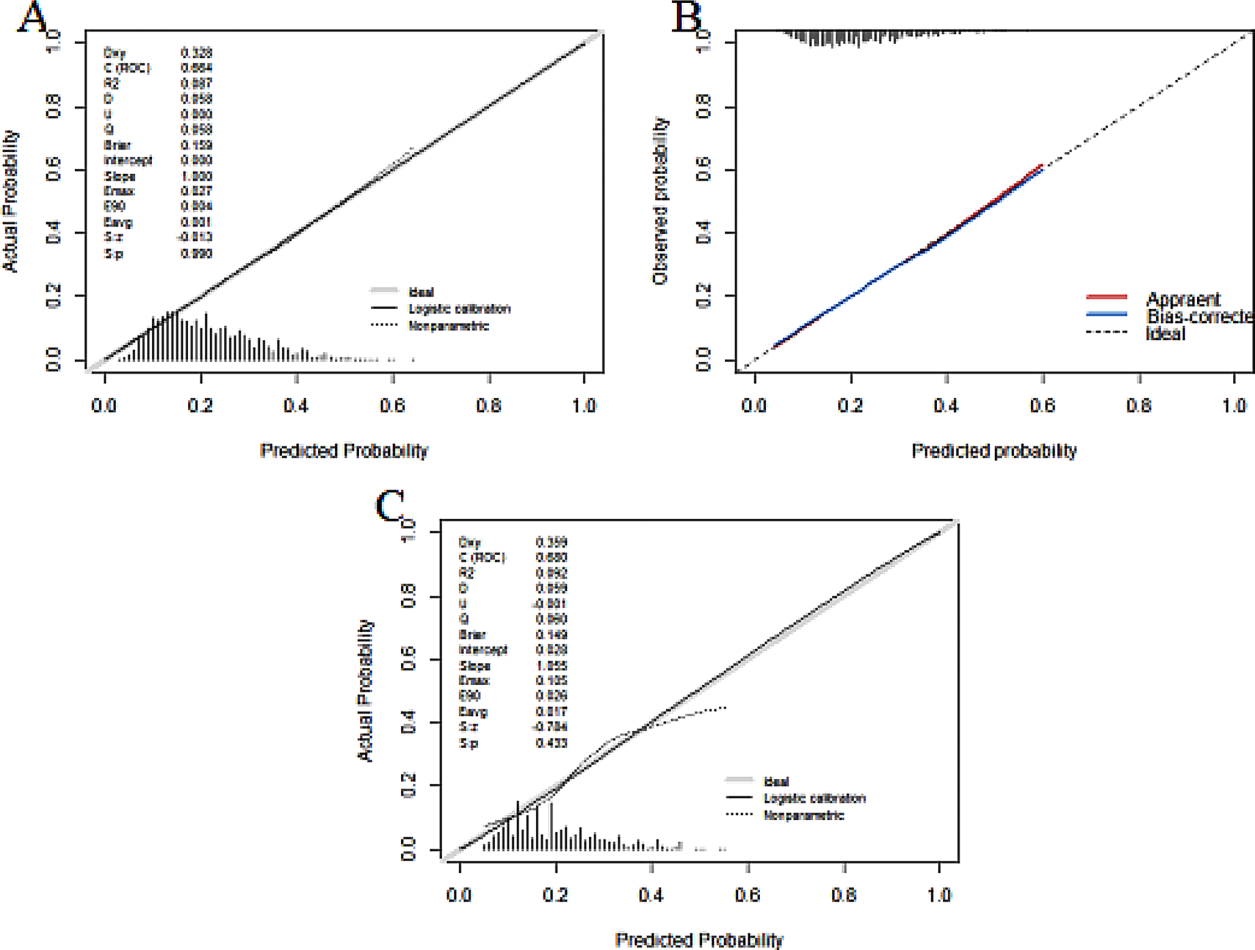
Calibration plots for the male modeling group (**A**), bootstrap internal validation group (**B**), and external validation group (**C**)

The predictive efficacy of the model was assessed and validated using ROC curves, calibration plots, and DCA curves. C-index of the modeling, external validation group according to the ROC curves, and bootstrap internal validation was 0.664 (95% CI: 0.652–0.676), 0.680 (95% CI: 0.647–0.712) (Fig. 2), and 0.660 (95% CI: 0.648–0.672) among male patients, respectively. The optimal cutoff points for the male modeling group and external validation group were 0.222 and 0.206, respectively, while those for the female modeling group and external validation group were 0.361 and 0.400(Fig. 2). In the calibration plots (Fig. 3) for the modeling group, internal validation group, and external validation group, the prediction curves were well-fitted to the reference line, demonstrating that the model-predicted risk was more consistent with the risk of actual occurrence of KOA. The DCA curves of the model in the mold-building group and external validation (Fig. 4) indicated that the net benefit of the model was higher than that of the all-treated population and the all-untreated population when the threshold probability was 0.1–0.4. This indicates that the model demonstrated the best clinical validity in this range and that the adoption of therapeutic measures exhibited a higher net benefit.

**Fig. 5:**
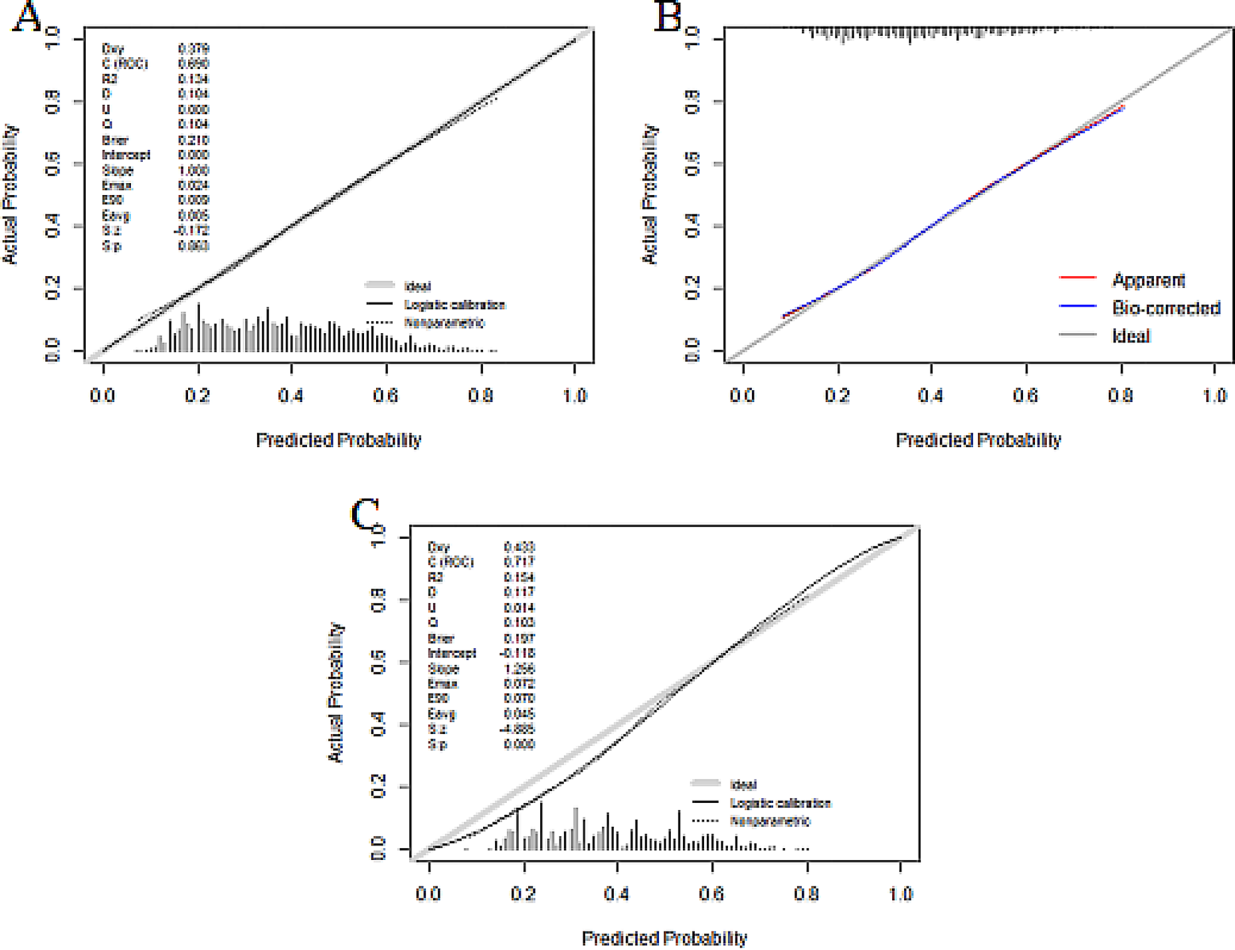
Calibration plots for the female modeling group (**A**), bootstrap internal validation group (**B**), and external validation group (**C**)

**Fig. 6:**
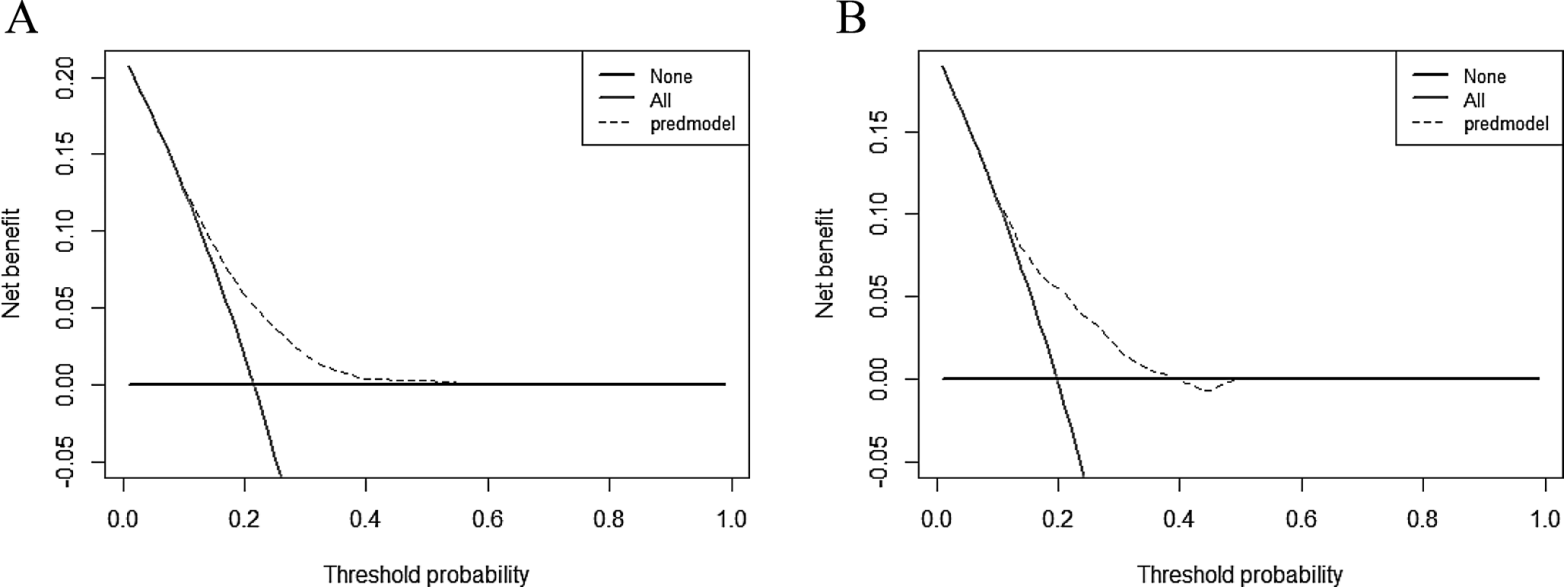
DCA curves for the male modeling group (**A**) and external validation (**B**)

**Fig. 7:**
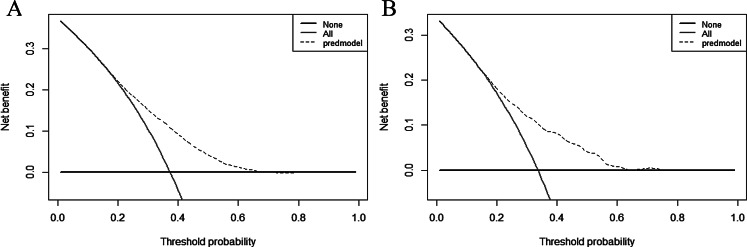
DCA curves for the female modeling group (**A**) and external validation (**B**)

The C-index of the modeling, external validation group, and Bootstrap internal validation was 0.690 (95% CI: 0.681–0.698), 0.716 (95% CI: 0.693–0.740) (Fig. 5), and 0.688 (95% CI: 0.679–0.696), respectively, among female patients according to the ROC curves. In the calibration plots of the modeling, internal validation, and external validation groups (Fig. 6), the prediction curves were well-fitted to the reference line, demonstrating that the model-predicted risk was more consistent with the risk of the actual KOA occurrence. The DCA curves of the model in the mold-building group and external validation (Fig. 7) indicated that the net benefit of the model was higher than that of the all-treated population and the all-untreated population when the threshold probability was 0.2–0.7. This indicates that the model demonstrated the best clinical validity in this range and that the adoption of therapeutic measures exhibited a higher net benefit.

**Fig. 8:**
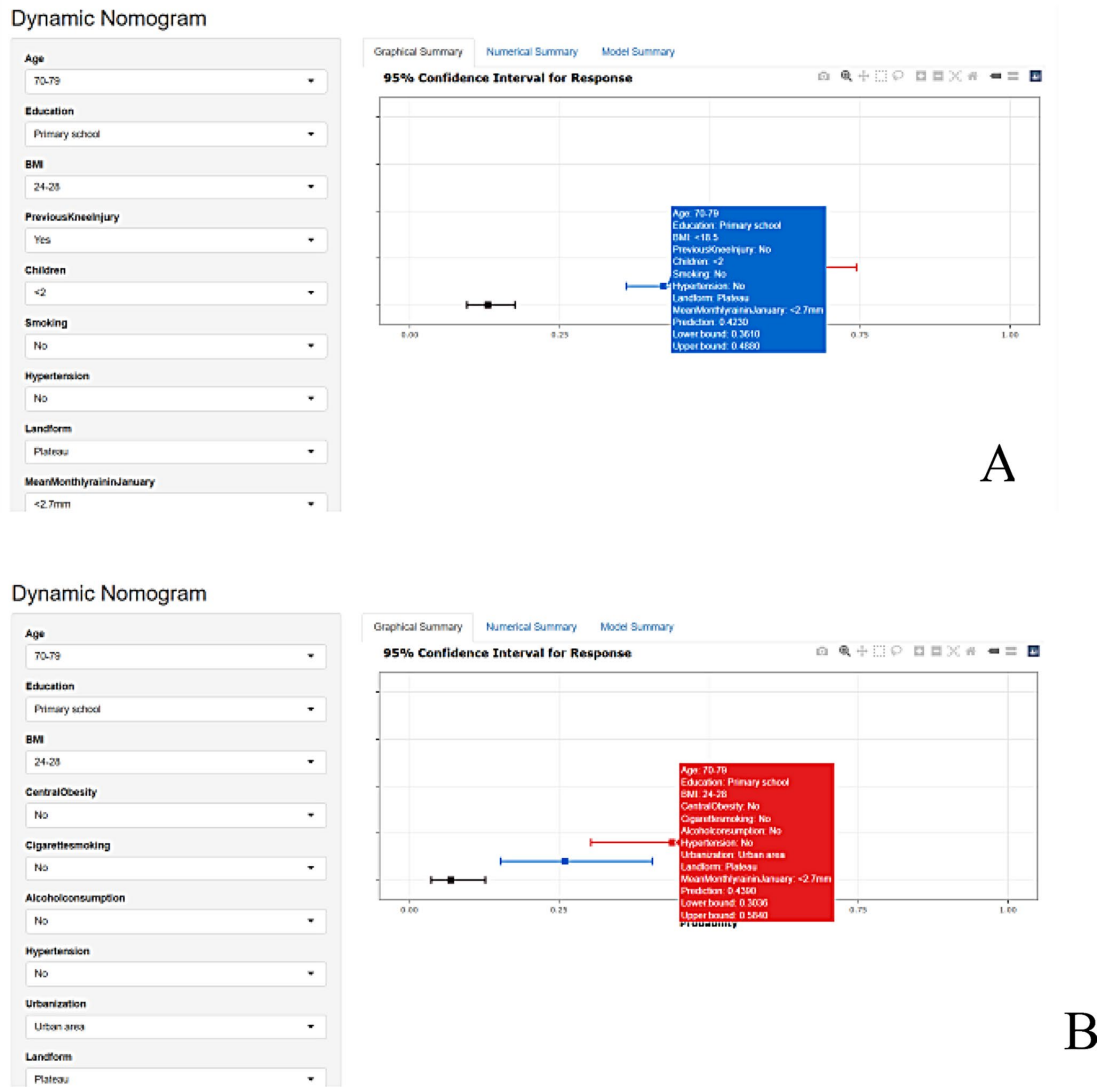
R shiny-based web interactive calculator application example for women (**A**) aand men (**B**). *This diagram demonstrates an example of using the interactive calculator based on R shiny. To calculate the probability of developing knee osteoarthritis, let's take women as an example. In the webpage shown in Figure (**A**), select clinical characteristics such as age (e.g., 70-79 years), education level (e.g., primary school), and BMI (e.g., <18.5kg/m²). Click the "predict" button to automatically calculate the probability of knee osteoarthritis (e.g., 0.423) along with its 95% confidence interval (e.g., 0.361-0.488). The process is identical for men: In the webpage shown in Figure (**B**), select corresponding characteristics and click "predict" to obtain results

An interactive web calculator based on R shiny was created by directly accessing web pages https://kneeosteoarthritisnomogram.shinyapps.io/DynNomapp/ (male) and https://femalekneeosteoarthritisnomogram.shinyapps.io/DynNomapp/ (female). It calculates the different genders of people suffering from knee arthritis probability and 95% CI by inputting clinical features. For instance, in a woman aged 70–79 years, with primary school education, BMI of < 18.5 kg/m^2^, no previous knee injury, < two children, no smoking, no hypertension, and living in the plateau, and < 2.7 mm average precipitation in January, you can select on the web page and click “predict” to automatically calculate the probability of developing knee arthritis at a probability of 0.423 (95% CI: 0.361–0.488). In a man aged 70–79 years, with primary school education, BMI of 24–28 kg/m^2^, non-central obesity, non-smoking, non-drinking, non-hypertension, living in urban areas, living in the plateau, and < 2.7 mm January average precipitation, you can click the “predict” button to automatically calculate the probability of suffering from knee arthritis, which was 0.439 (95% CI: 0.304–0.584, Fig. 8).

## Discussion

CNKS is a national KOA epidemiological survey. We revealed that the population-weighted prevalence of radiological KOA in China was 27.9 (95% CI: 24.8–31.1) per 1000 population, which extrapolates to an estimated 38.5 million individuals (34.2–42.9 million people) with radiological KOA in China. The results of this study indicate radiological KOA as a major public health problem in modern China. However, comparison of our results with those from other countries is challenging. Population-based studies on the prevalence of radiological KOA have been conducted in some regions; however, not all regions have reported consistent results. Epidemiological differences between populations are important because they imply different cultures and lifestyles in each region. For instance, a 2020 survey of 12,287 people in South Korea by Hong JW et al. demonstrated a 35.1% prevalence of radiological KOA by X-imaging in the adult population of South Korea [13].

Using data collected from the CNKS, which is a national population survey, we observed that symptomatic KOA was prevalent among Chinese adults, especially among those with lower socioeconomic status. The remarkable variation in the prevalence of symptomatic KOA based on geographic location indicates a much higher prevalence among residents living in the southwest region than among those living in other parts of China. In our study, the prevalence of symptomatic KOA (8.1%) was higher than that in the Framingham OA study (6.7% of those age s45 years) [14]especially among women (10.3% in China versus 7.2% in Framingham, MA). The prevalence of coronavirus disease 2019 (COVID-19) in China was also higher than that among individuals in European countries [15]. In contrast, the prevalence of symptomatic KOA was much lower in the CNKS than in the Johnston County OA Project (16.4%) [16]. We postulate that this disparity may be partly associated with the difference in the prevalence of overweight and obesity between the two studies. In the Johnston County OA Project, 39.5% of participants were overweight and 34.3% were obese. In contrast, 25.9% of the Chinese participants were overweight and 4.9% were obese. Several studies [17–19] have revealed a higher prevalence of symptomatic KOA in women and increased with age. The results of our study corroborated these findings. Interestingly, the prevalence of symptomatic KOA increased with age until age 70 years and then leveled off. Such a phenomenon was also observed in other studies. We speculate that elderly adults may have reduced their engagement level in heavy physical activity, thereby decreasing knee symptoms. Further, previous studies have demonstrated that symptomatic KOA is associated with higher mortality [20,21]. Thus, individuals with symptomatic KOA may have died prematurely, thereby causing the reported lower prevalence of symptomatic KOA than the actual prevalence. In several studies, socioeconomic status was associated with symptomatic KOA [22, 23]. For instance, the prevalence of symptomatic KOA was higher among participants who received less education than among those who received more education [24] and among those who lived in communities with high poverty rates [25]. Our study also revealed that symptomatic KOA was more prevalent among persons living in rural areas than among those living in urban areas in China. This result was consistent with the findings of previous studies in which the prevalence of symptomatic KOA may have been underreported. However, considering the already high prevalence of symptomatic KOA in those areas, the real differences between regions could be higher than those reported in the current study. In conclusion, the prevalence of symptomatic KOA in China was high using data from the CNKS. The prevalence varied significantly according to sociodemographic and economic factors and was much higher in women, older individuals, and persons living in underdeveloped areas.

In this study, a predictive model for KOA was developed by analyzing large-scale data, and its validity was verified in male and female patients. The results indicate that age, education, BMI, central obesity, and hypertension were significant risk factors for KOA, whereas smoking, alcohol consumption, living in urban and highland areas, and higher average January precipitation were protective factors. These findings are consistent with those of the existing literature. Age is a crucial risk factor for KOA. The articular cartilage gradually deteriorates with age, causing increased KOA incidence. In the present study, the risk of KOA was significantly increased in people aged 60 years, which is consistent with the results of Felson et al. [26, 27]. Individuals with lower education levels are more likely to develop KOA, which may be associated with their lack of health awareness and unhealthy lifestyles [28, 29]. In this study, we revealed a significantly increased risk of KOA in people who attained junior high school and lower education, which is consistent with the findings of Jordan et al. High BMI is an important risk factor for KOA. In this study, the risk of KOA was significantly increased in those with a BMI of ≥ 24 kg/m², consistent with the results of Blagojevic et al. Obesity increases the mechanical load on the knee joint and accelerates articular cartilage degradation [30, 31]. This has been further supported by studies in recent years, reporting that obesity not only affects the joint through mechanical loading but may also increase the inflammatory response through metabolic pathways [32, 33]. Hypertension is closely associated with KOA development. Hypertension may promote KOA development by affecting the blood supply to the joints and increasing the inflammatory response [34]. Further, patients who take long-term anti-hypertensive and hypoglycemic medications demonstrated a higher risk of KOA, which may be associated with the side effects of the medications [35].

Interestingly, our study revealed smoking as a protective factor for KOA, which contradicts the results of some studies [36]. However, other studies have indicated that smoking reduces KOA by suppressing the inflammatory response [37]. The protective effects of alcohol consumption may be associated with the benefits of moderate alcohol consumption on cardiovascular health [38]. The residence of men in hilly areas is a risk factor for developing KOA, and the risk of KOA is lower in people living in urban and highland areas, which may be associated with climatic conditions and lifestyles in these areas [39]. Our study revealed that higher average precipitation in January was also a protective factor against KOA, which may be related to the lubricating effect of humidity on the joints. Further, for women, previous knee damage and ≥ 2 childbirths are risk factors for developing KOA. Early studies have demonstrated that knee injuries, especially severe injuries, such as meniscal tears or anterior cruciate ligament ruptures, are strongly associated with the development of future KOA [40]. Gelber et al. revealed that the cumulative incidence of KOA at the age of 65 years was 13.9% in participants with knee injuries in adolescence and early adulthood and 6.0% in uninjured subjects. Young adults with knee injuries are at a greatly increased risk of developing KOA later in life [41]. Childbirth is a crucial experience in a woman’s life, and multiple births may have some effect on a woman’s body. Liu et al. revealed that the risk of knee arthroplasty increased by 8% for each additional birth [42]. Childbirth may affect the occurrence and development of KOA in women through various pathways. Estrogen levels significantly change during childbirth. Further, multiple births cause weight gain, and being overweight or obese is one of the most important risk factors for KOA. Lifestyle changes occur after childbirth, and women may be less physically active due to childcare, which may increase the risk of developing KOA [43].

In this study, a predictive model for KOA was developed through large-scale data analysis, which provides clinicians with an effective tool for determining high-risk populations and taking early intervention measures. This approach is important for improving the quality of life of patients with KOA. The KOA prediction model developed in this study exhibited high predictive efficacy in both men and women, demonstrating important clinical applications. By the early identification of high-risk groups, KOA can be better prevented and managed, and the quality of life of patients can be improved. However, this study has some limitations such as geographical bias in the data and the omission of potential influencing factors. Future studies are recommended to further validate the applicability of the model in different populations and explore more possible predictors. Further, the inclusion of more biomarkers and genetic information should be considered to improve the predictive accuracy of the model [44].

In summary, the CNKS was a detailed epidemiological survey conducted nationwide to assess the prevalence of KOA among the population. The current research provides detailed information on the population-weighted prevalence and distribution of radioactive KOA in China, establishes a KOA risk prediction model, identifies risk factors for symptomatic KOA, and predicts its occurrence probability. These pieces of information can serve as the latest clinical evidence basis for China’s national healthcare planning and prevention efforts to develop public health policies with a focus on preventing hypertension, reducing fertility, and maintaining a healthy weight to help reduce KOA risk. Health education on KOA needs to be strengthened and lifestyle changes should be promoted, especially among the elderly, those with junior high school education or below, and those with a history of knee joint injuries, to reduce the risk of KOA.

## Conclusions

This is the first national KOA survey in China to identify distinct gender-specific risk factors. The validated prediction models facilitate early risk stratification, supporting targeted public health interventions that focus on weight management, hypertension control, and terrain-adapted prevention strategies. Implementation of our web-based tools in primary care may reduce the increasing KOA burden among aging populations.

## Supplementary Information

Below is the link to the electronic supplementary material.

Supplementary Material 1

Supplementary Material 2

Supplementary Material 3

## Data Availability

Data is provided within the manuscript or supplementary information files.

## Declarations

### Disclose the use of AI and AI-assisted technologies

Not used.
