# Supplementary material for: Prevalence of radiographic knee osteoarthritis in China: a national survey of thirty thousand, four hundred and fifty five individuals cross-sectional study
Source: Int Orthop. 2025 Sep 3;49(10):2489–507. doi: 10.1007/s00264-025-06643-9 (PMC12488765; doi:10.1007/s00264-025-06643-9)
Supplement: Supplementary file 1 — Supplementary Material 1 [file 264_2025_6643_MOESM1_ESM.docx]

**Appendix I**

**Classification of Central Obesity, Smoking and Other Demographic Variables Utilised in the Chinese National KOA Study (CNKS)**

**Age Groups**

There were 4 age range categories used in this study: 50-59 years, 60-69 years, 70-79 years, and ≥80 years.

**Ethnicity**

Ethnicity was classified as either the Han nationality or other nationalities.

**Geographical Region**

Three geographical regions were used in this study: east, central and west.

**Residential Category**

Two regions were used in this study: urban and rural areas.

**Landform Category**

Four regions were used in this study: plain, mountainous areas, plateau, basin.

**Coastal or Innerland**

Two regions were used in this study: coastal and innerland.

**Occupation**

There were 8 occupational categories: officer, office clerk, technician, business and service personnel, farmer, manual worker and transportation personnel, unemployed, and other occupations.^1^

**Education**

Four levels were utilized for the classification of highest educational attainment in this study: 1) illiterate, 2) primary school, 3) junior high school, 4) senior high school, 5) College or above. Illiteracy was defined as adults who were unable to read and write.

**Alcohol Consumption**

Alcohol consumption was defined as the consumption of at least one standard alcoholic drink more than once per week for at least 6 months.^2^ An alcoholic drink was defined as a can or bottle of beer, a glass of wine, a small glass of port/sherry, or a nip of spirits/liquor. Total cumulative alcohol consumption to date was classified as <300 kg, 300-599 kg and >600 kg.

**Cigarette Smoking**

Participants who had smoked more than 10 cigarettes per week for at least 6 months prior to the survey were defined as cigarette smokers.^2^ Total cumulative smoking to date was classified as <100k, 100 - 299k and >300k.

**Calcium or Vitamin D Supplement**

Routine use of nonprescription calcium or Vitamin D supplements or prescription calcium or Vitamin D supplements according to a physicians' therapeutic recommendation within the 6 months prior to the fracture occurrence (for those with fractures) or answering on their questionnaire (for those without fractures).

**Body Mass Index (BMI) Category**

There were four BMI categories used in this study: <18.5kg/m^2^ (underweight), 18.5-23.9kg/m^2^ (normal), 24-27.9 kg/m^2^ (overweight), and ≥28kg/m^2^ (obese).^3^

**Waist circumference**

There were three waist circumference categories used in this study: <90 cm (normal), 90-100 cm (overweight risk), >100 cm (obese risk) for male and <85 cm (normal), 85-95 cm (overweight risk), >95 cm (obese risk) for female. Central obesity was defined as ≥90cm for male and ≥85cm for female.

**Born in Famine years**

Participants born between 1959 and 1961 were defined as born in famine years.

**Per capita month income**

Three levels were used in this study: <1000 RMB/month, 1000-1999 RMB/month, >2000 RMB/month.

**Number of Children**

Participants gave birth to several children in her lifetime for three levels in this study: ≤1, 2, ≥3

**Menopause age**

Three levels were used in this study: ≤45 years, 46-50 years, >50 years.

**Mean Monthly income**

Per capita monthly household income of participants: <1000 RMB/month, 1000-1999 RMB/month, >2000 RMB/month.

**References**

1. Wang LD, Qi XQ, Chen CH, Ma JK, Li LM, Rao KQ (Eds). The 2002 comprehensive report on the nutrition and health status of Chinese residents (1^st^Edition) [in Chinese]. Beijing, China: People's Medical Publishing House, 2005.

2.[Tai SY](https://www.ncbi.nlm.nih.gov/pubmed/?term=Tai%20SY%5BAuthor%5D&cauthor=true&cauthor_uid=20333794),[Wu IC](https://www.ncbi.nlm.nih.gov/pubmed/?term=Wu%20IC%5BAuthor%5D&cauthor=true&cauthor_uid=20333794),[Wu DC](https://www.ncbi.nlm.nih.gov/pubmed/?term=Wu%20DC%5BAuthor%5D&cauthor=true&cauthor_uid=20333794),[Su HJ](https://www.ncbi.nlm.nih.gov/pubmed/?term=Su%20HJ%5BAuthor%5D&cauthor=true&cauthor_uid=20333794),[Huang JL](https://www.ncbi.nlm.nih.gov/pubmed/?term=Huang%20JL%5BAuthor%5D&cauthor=true&cauthor_uid=20333794),[Tsai HJ](https://www.ncbi.nlm.nih.gov/pubmed/?term=Tsai%20HJ%5BAuthor%5D&cauthor=true&cauthor_uid=20333794),[Lu CY](https://www.ncbi.nlm.nih.gov/pubmed/?term=Lu%20CY%5BAuthor%5D&cauthor=true&cauthor_uid=20333794),[Lee JM](https://www.ncbi.nlm.nih.gov/pubmed/?term=Lee%20JM%5BAuthor%5D&cauthor=true&cauthor_uid=20333794),[Wu MT](https://www.ncbi.nlm.nih.gov/pubmed/?term=Wu%20MT%5BAuthor%5D&cauthor=true&cauthor_uid=20333794). Cigarette smoking and alcohol drinking and esophageal cancer risk in Taiwanese women. [World J Gastroenterol](https://www.ncbi.nlm.nih.gov/pubmed/20333794) 2010;16(12):1518-21.

3. Criteria for the Weight of Adults. National Health and Family Planning Commission of the People's Republic of China (WS/T 428-2013, <http://www.nhfpc.gov.cn/ewebeditor/uploadfile/2013/08/20130808135715967.pdf>)[in Chinese].
