# Supplementary material for: Prevalence of radiographic knee osteoarthritis in China: a national survey of thirty thousand, four hundred and fifty five individuals cross-sectional study
Source: Int Orthop. 2025 Sep 3;49(10):2489–507. doi: 10.1007/s00264-025-06643-9 (PMC12488765; doi:10.1007/s00264-025-06643-9)
Supplement: Supplementary file 2 — Supplementary Material 2 [file 264_2025_6643_MOESM2_ESM.docx]

**Appendix Ⅱ**

**Correlations of Central Obesity, Smoking and Each Variable in the Chinese National KOA Study (CNKS)**

| ISSU | | Age | Ethnicity | Education | Born in Famine years | BMI | Central Obesity | Previous Knee Injury | Children | Smoking | Alcohol | VD or calcium | Hypertension | Diabetes | digestive drug | anti-hypertensive drug | anti-diabetic drugs | Urbanization | Landform | Region | income | Mean Monthly Temperature in January（℃） | Mean Monthly rain in January(mm) | Mean Monthly Humidity in January（%） | Mean Monthly rain in July(mm) | Mean Monthly Humidity in July（%） | Mean Monthly Temperature in July（℃） |
| --- | --- | --- | --- | --- | --- | --- | --- | --- | --- | --- | --- | --- | --- | --- | --- | --- | --- | --- | --- | --- | --- | --- | --- | --- | --- | --- | --- |
| Age | Pearson Correlation | 1 | -.017^*^ | -.345^**^ | -.313^**^ | -.064^**^ | .019^*^ | -.016^*^ | .475^**^ | 0.001 | -.025^**^ | -0.005 | .140^**^ | .058^**^ | .072^**^ | .128^**^ | .058^**^ | 0.009 | .017^*^ | -.064^**^ | -.089^**^ | .079^**^ | .052^**^ | .036^**^ | .038^**^ | 0.002 | .065^**^ |
|  | Sig. (2-tailed) |  | 0.031 | 0.000 | 0.000 | 0.000 | 0.011 | 0.033 | 0.000 | 0.904 | 0.001 | 0.548 | 0.000 | 0.000 | 0.000 | 0.000 | 0.000 | 0.255 | 0.028 | 0.000 | 0.000 | 0.000 | 0.000 | 0.000 | 0.000 | 0.796 | 0.000 |
|  | N | 17011 | 17011 | 17011 | 17011 | 17011 | 17011 | 17011 | 17011 | 17011 | 17011 | 17011 | 17011 | 17011 | 17011 | 17011 | 17011 | 17011 | 17011 | 17011 | 17011 | 17011 | 17011 | 17011 | 17011 | 17011 | 17011 |
| Ethnicity | Pearson Correlation | -.017^*^ | 1 | 0.013 | -0.009 | .021^**^ | .045^**^ | 0.005 | -0.002 | 0.004 | 0.010 | -0.003 | 0.006 | 0.000 | 0.002 | 0.010 | 0.001 | 0.011 | .221^**^ | .096^**^ | -.017^*^ | -.158^**^ | -.079^**^ | -0.010 | -.191^**^ | -.298^**^ | -.153^**^ |
|  | Sig. (2-tailed) | 0.031 |  | 0.100 | 0.234 | 0.006 | 0.000 | 0.536 | 0.769 | 0.601 | 0.195 | 0.657 | 0.417 | 0.952 | 0.788 | 0.188 | 0.851 | 0.144 | 0.000 | 0.000 | 0.026 | 0.000 | 0.000 | 0.197 | 0.000 | 0.000 | 0.000 |
|  | N | 17011 | 17011 | 17011 | 17011 | 17011 | 17011 | 17011 | 17011 | 17011 | 17011 | 17011 | 17011 | 17011 | 17011 | 17011 | 17011 | 17011 | 17011 | 17011 | 17011 | 17011 | 17011 | 17011 | 17011 | 17011 | 17011 |
| Education | Pearson Correlation | -.345^**^ | 0.013 | 1 | .135^**^ | .026^**^ | -.035^**^ | .036^**^ | -.357^**^ | 0.010 | 0.011 | .034^**^ | -.044^**^ | -0.004 | -0.012 | -.049^**^ | -.019^*^ | .156^**^ | .046^**^ | .126^**^ | .245^**^ | -.208^**^ | -.179^**^ | -.130^**^ | -.166^**^ | -.109^**^ | -.188^**^ |
|  | Sig. (2-tailed) | 0.000 | 0.100 |  | 0.000 | 0.001 | 0.000 | 0.000 | 0.000 | 0.208 | 0.137 | 0.000 | 0.000 | 0.605 | 0.107 | 0.000 | 0.013 | 0.000 | 0.000 | 0.000 | 0.000 | 0.000 | 0.000 | 0.000 | 0.000 | 0.000 | 0.000 |
|  | N | 17011 | 17011 | 17011 | 17011 | 17011 | 17011 | 17011 | 17011 | 17011 | 17011 | 17011 | 17011 | 17011 | 17011 | 17011 | 17011 | 17011 | 17011 | 17011 | 17011 | 17011 | 17011 | 17011 | 17011 | 17011 | 17011 |
| Born in Famine years | Pearson Correlation | -.313^**^ | -0.009 | .135^**^ | 1 | 0.011 | -.016^*^ | -0.007 | -.156^**^ | 0.007 | 0.005 | .027^**^ | -.030^**^ | -0.015 | -.021^**^ | -.030^**^ | -.016^*^ | .037^**^ | .027^**^ | -0.014 | .022^**^ | -0.015 | 0.000 | -0.006 | -.018^*^ | -0.006 | -.022^**^ |
|  | Sig. (2-tailed) | 0.000 | 0.234 | 0.000 |  | 0.162 | 0.038 | 0.379 | 0.000 | 0.341 | 0.491 | 0.000 | 0.000 | 0.056 | 0.007 | 0.000 | 0.034 | 0.000 | 0.000 | 0.075 | 0.004 | 0.055 | 0.969 | 0.451 | 0.018 | 0.435 | 0.004 |
|  | N | 17011 | 17011 | 17011 | 17011 | 17011 | 17011 | 17011 | 17011 | 17011 | 17011 | 17011 | 17011 | 17011 | 17011 | 17011 | 17011 | 17011 | 17011 | 17011 | 17011 | 17011 | 17011 | 17011 | 17011 | 17011 | 17011 |
| BMI | Pearson Correlation | -.064^**^ | .021^**^ | .026^**^ | 0.011 | 1 | .522^**^ | .044^**^ | -0.004 | -0.014 | 0.007 | .018^*^ | .198^**^ | .062^**^ | 0.000 | .186^**^ | .057^**^ | -0.002 | .030^**^ | .110^**^ | 0.013 | -.108^**^ | -.146^**^ | -.163^**^ | -.144^**^ | -.133^**^ | -.062^**^ |
|  | Sig. (2-tailed) | 0.000 | 0.006 | 0.001 | 0.162 |  | 0.000 | 0.000 | 0.584 | 0.064 | 0.351 | 0.019 | 0.000 | 0.000 | 0.984 | 0.000 | 0.000 | 0.771 | 0.000 | 0.000 | 0.081 | 0.000 | 0.000 | 0.000 | 0.000 | 0.000 | 0.000 |
|  | N | 17011 | 17011 | 17011 | 17011 | 17011 | 17011 | 17011 | 17011 | 17011 | 17011 | 17011 | 17011 | 17011 | 17011 | 17011 | 17011 | 17011 | 17011 | 17011 | 17011 | 17011 | 17011 | 17011 | 17011 | 17011 | 17011 |
| Central Obesity | Pearson Correlation | .019^*^ | .045^**^ | -.035^**^ | -.016^*^ | .522^**^ | 1 | .038^**^ | .036^**^ | 0.001 | -0.008 | 0.006 | .209^**^ | .089^**^ | .015^*^ | .204^**^ | .083^**^ | -.037^**^ | .061^**^ | .139^**^ | -0.013 | -.140^**^ | -.212^**^ | -.202^**^ | -.180^**^ | -.173^**^ | -.080^**^ |
|  | Sig. (2-tailed) | 0.011 | 0.000 | 0.000 | 0.038 | 0.000 |  | 0.000 | 0.000 | 0.864 | 0.299 | 0.420 | 0.000 | 0.000 | 0.044 | 0.000 | 0.000 | 0.000 | 0.000 | 0.000 | 0.090 | 0.000 | 0.000 | 0.000 | 0.000 | 0.000 | 0.000 |
|  | N | 17011 | 17011 | 17011 | 17011 | 17011 | 17011 | 17011 | 17011 | 17011 | 17011 | 17011 | 17011 | 17011 | 17011 | 17011 | 17011 | 17011 | 17011 | 17011 | 17011 | 17011 | 17011 | 17011 | 17011 | 17011 | 17011 |
| Previous Knee Injury | Pearson Correlation | -.016^*^ | 0.005 | .036^**^ | -0.007 | .044^**^ | .038^**^ | 1 | -.018^*^ | .031^**^ | .020^**^ | .023^**^ | .021^**^ | .015^*^ | -0.002 | .019^*^ | 0.003 | .032^**^ | .039^**^ | .032^**^ | 0.000 | -.057^**^ | -.051^**^ | -.027^**^ | -.040^**^ | -.029^**^ | -.054^**^ |
|  | Sig. (2-tailed) | 0.033 | 0.536 | 0.000 | 0.379 | 0.000 | 0.000 |  | 0.020 | 0.000 | 0.009 | 0.003 | 0.006 | 0.049 | 0.787 | 0.014 | 0.729 | 0.000 | 0.000 | 0.000 | 0.978 | 0.000 | 0.000 | 0.000 | 0.000 | 0.000 | 0.000 |
|  | N | 17011 | 17011 | 17011 | 17011 | 17011 | 17011 | 17011 | 17011 | 17011 | 17011 | 17011 | 17011 | 17011 | 17011 | 17011 | 17011 | 17011 | 17011 | 17011 | 17011 | 17011 | 17011 | 17011 | 17011 | 17011 | 17011 |
| Children | Pearson Correlation | .475^**^ | -0.002 | -.357^**^ | -.156^**^ | -0.004 | .036^**^ | -.018^*^ | 1 | -.031^**^ | -.040^**^ | -0.001 | .090^**^ | .028^**^ | .036^**^ | .091^**^ | .042^**^ | -.185^**^ | -.017^*^ | .020^**^ | -.170^**^ | .182^**^ | -.023^**^ | .031^**^ | .111^**^ | .021^**^ | .154^**^ |
|  | Sig. (2-tailed) | 0.000 | 0.769 | 0.000 | 0.000 | 0.584 | 0.000 | 0.020 |  | 0.000 | 0.000 | 0.866 | 0.000 | 0.000 | 0.000 | 0.000 | 0.000 | 0.000 | 0.026 | 0.008 | 0.000 | 0.000 | 0.003 | 0.000 | 0.000 | 0.006 | 0.000 |
|  | N | 17011 | 17011 | 17011 | 17011 | 17011 | 17011 | 17011 | 17011 | 17011 | 17011 | 17011 | 17011 | 17011 | 17011 | 17011 | 17011 | 17011 | 17011 | 17011 | 17011 | 17011 | 17011 | 17011 | 17011 | 17011 | 17011 |
| Smoking | Pearson Correlation | 0.001 | 0.004 | 0.010 | 0.007 | -0.014 | 0.001 | .031^**^ | -.031^**^ | 1 | .233^**^ | 0.009 | 0.014 | 0.009 | 0.003 | 0.011 | -0.002 | .036^**^ | .052^**^ | 0.005 | 0.005 | -.083^**^ | -.038^**^ | 0.004 | 0.004 | 0.014 | -.104^**^ |
|  | Sig. (2-tailed) | 0.904 | 0.601 | 0.208 | 0.341 | 0.064 | 0.864 | 0.000 | 0.000 |  | 0.000 | 0.253 | 0.071 | 0.263 | 0.719 | 0.152 | 0.762 | 0.000 | 0.000 | 0.475 | 0.518 | 0.000 | 0.000 | 0.608 | 0.605 | 0.063 | 0.000 |
|  | N | 17011 | 17011 | 17011 | 17011 | 17011 | 17011 | 17011 | 17011 | 17011 | 17011 | 17011 | 17011 | 17011 | 17011 | 17011 | 17011 | 17011 | 17011 | 17011 | 17011 | 17011 | 17011 | 17011 | 17011 | 17011 | 17011 |
| Alcohol | Pearson Correlation | -.025^**^ | 0.010 | 0.011 | 0.005 | 0.007 | -0.008 | .020^**^ | -.040^**^ | .233^**^ | 1 | 0.011 | -0.002 | -0.003 | -0.004 | -0.007 | -0.014 | .019^*^ | 0.001 | 0.000 | .020^**^ | -.027^**^ | .028^**^ | .034^**^ | 0.010 | .035^**^ | -.023^**^ |
|  | Sig. (2-tailed) | 0.001 | 0.195 | 0.137 | 0.491 | 0.351 | 0.299 | 0.009 | 0.000 | 0.000 |  | 0.134 | 0.756 | 0.667 | 0.615 | 0.336 | 0.078 | 0.016 | 0.869 | 0.952 | 0.010 | 0.000 | 0.000 | 0.000 | 0.204 | 0.000 | 0.003 |
|  | N | 17011 | 17011 | 17011 | 17011 | 17011 | 17011 | 17011 | 17011 | 17011 | 17011 | 17011 | 17011 | 17011 | 17011 | 17011 | 17011 | 17011 | 17011 | 17011 | 17011 | 17011 | 17011 | 17011 | 17011 | 17011 | 17011 |
| VD or calcium | Pearson Correlation | -0.005 | -0.003 | .034^**^ | .027^**^ | .018^*^ | 0.006 | .023^**^ | -0.001 | 0.009 | 0.011 | 1 | .017^*^ | -0.002 | -0.004 | .016^*^ | -0.008 | .032^**^ | .067^**^ | 0.007 | -0.007 | -.017^*^ | -.033^**^ | -.031^**^ | -.044^**^ | -.045^**^ | -.037^**^ |
|  | Sig. (2-tailed) | 0.548 | 0.657 | 0.000 | 0.000 | 0.019 | 0.420 | 0.003 | 0.866 | 0.253 | 0.134 |  | 0.030 | 0.760 | 0.586 | 0.034 | 0.273 | 0.000 | 0.000 | 0.376 | 0.380 | 0.024 | 0.000 | 0.000 | 0.000 | 0.000 | 0.000 |
|  | N | 17011 | 17011 | 17011 | 17011 | 17011 | 17011 | 17011 | 17011 | 17011 | 17011 | 17011 | 17011 | 17011 | 17011 | 17011 | 17011 | 17011 | 17011 | 17011 | 17011 | 17011 | 17011 | 17011 | 17011 | 17011 | 17011 |
| Hypertension | Pearson Correlation | .140^**^ | 0.006 | -.044^**^ | -.030^**^ | .198^**^ | .209^**^ | .021^**^ | .090^**^ | 0.014 | -0.002 | .017^*^ | 1 | .169^**^ | .040^**^ | .884^**^ | .145^**^ | .032^**^ | .058^**^ | .095^**^ | -0.013 | -.105^**^ | -.094^**^ | -.124^**^ | -.135^**^ | -.109^**^ | -.070^**^ |
|  | Sig. (2-tailed) | 0.000 | 0.417 | 0.000 | 0.000 | 0.000 | 0.000 | 0.006 | 0.000 | 0.071 | 0.756 | 0.030 |  | 0.000 | 0.000 | 0.000 | 0.000 | 0.000 | 0.000 | 0.000 | 0.101 | 0.000 | 0.000 | 0.000 | 0.000 | 0.000 | 0.000 |
|  | N | 17011 | 17011 | 17011 | 17011 | 17011 | 17011 | 17011 | 17011 | 17011 | 17011 | 17011 | 17011 | 17011 | 17011 | 17011 | 17011 | 17011 | 17011 | 17011 | 17011 | 17011 | 17011 | 17011 | 17011 | 17011 | 17011 |
| Diabetes | Pearson Correlation | .058^**^ | 0.000 | -0.004 | -0.015 | .062^**^ | .089^**^ | .015^*^ | .028^**^ | 0.009 | -0.003 | -0.002 | .169^**^ | 1 | 0.005 | .177^**^ | .891^**^ | .042^**^ | .048^**^ | .049^**^ | .016^*^ | -.077^**^ | -.049^**^ | -.025^**^ | -.065^**^ | -.052^**^ | -.052^**^ |
|  | Sig. (2-tailed) | 0.000 | 0.952 | 0.605 | 0.056 | 0.000 | 0.000 | 0.049 | 0.000 | 0.263 | 0.667 | 0.760 | 0.000 |  | 0.541 | 0.000 | 0.000 | 0.000 | 0.000 | 0.000 | 0.038 | 0.000 | 0.000 | 0.001 | 0.000 | 0.000 | 0.000 |
|  | N | 17011 | 17011 | 17011 | 17011 | 17011 | 17011 | 17011 | 17011 | 17011 | 17011 | 17011 | 17011 | 17011 | 17011 | 17011 | 17011 | 17011 | 17011 | 17011 | 17011 | 17011 | 17011 | 17011 | 17011 | 17011 | 17011 |
| digestive drug | Pearson Correlation | .072^**^ | 0.002 | -0.012 | -.021^**^ | 0.000 | .015^*^ | -0.002 | .036^**^ | 0.003 | -0.004 | -0.004 | .040^**^ | 0.005 | 1 | .034^**^ | -0.004 | .027^**^ | 0.011 | .029^**^ | .018^*^ | -.034^**^ | 0.008 | -0.001 | -.022^**^ | -.016^*^ | -.028^**^ |
|  | Sig. (2-tailed) | 0.000 | 0.788 | 0.107 | 0.007 | 0.984 | 0.044 | 0.787 | 0.000 | 0.719 | 0.615 | 0.586 | 0.000 | 0.541 |  | 0.000 | 0.567 | 0.000 | 0.163 | 0.000 | 0.018 | 0.000 | 0.321 | 0.941 | 0.004 | 0.038 | 0.000 |
|  | N | 17011 | 17011 | 17011 | 17011 | 17011 | 17011 | 17011 | 17011 | 17011 | 17011 | 17011 | 17011 | 17011 | 17011 | 17011 | 17011 | 17011 | 17011 | 17011 | 17011 | 17011 | 17011 | 17011 | 17011 | 17011 | 17011 |
| anti-hypertensive drug | Pearson Correlation | .128^**^ | 0.010 | -.049^**^ | -.030^**^ | .186^**^ | .204^**^ | .019^*^ | .091^**^ | 0.011 | -0.007 | .016^*^ | .884^**^ | .177^**^ | .034^**^ | 1 | .163^**^ | 0.008 | .057^**^ | .100^**^ | -.024^**^ | -.100^**^ | -.093^**^ | -.124^**^ | -.144^**^ | -.123^**^ | -.051^**^ |
|  | Sig. (2-tailed) | 0.000 | 0.188 | 0.000 | 0.000 | 0.000 | 0.000 | 0.014 | 0.000 | 0.152 | 0.336 | 0.034 | 0.000 | 0.000 | 0.000 |  | 0.000 | 0.309 | 0.000 | 0.000 | 0.002 | 0.000 | 0.000 | 0.000 | 0.000 | 0.000 | 0.000 |
|  | N | 17011 | 17011 | 17011 | 17011 | 17011 | 17011 | 17011 | 17011 | 17011 | 17011 | 17011 | 17011 | 17011 | 17011 | 17011 | 17011 | 17011 | 17011 | 17011 | 17011 | 17011 | 17011 | 17011 | 17011 | 17011 | 17011 |
| anti-diabetic drugs | Pearson Correlation | .058^**^ | 0.001 | -.019^*^ | -.016^*^ | .057^**^ | .083^**^ | 0.003 | .042^**^ | -0.002 | -0.014 | -0.008 | .145^**^ | .891^**^ | -0.004 | .163^**^ | 1 | .017^*^ | .035^**^ | .049^**^ | 0.012 | -.054^**^ | -.037^**^ | -.034^**^ | -.065^**^ | -.065^**^ | -.024^**^ |
|  | Sig. (2-tailed) | 0.000 | 0.851 | 0.013 | 0.034 | 0.000 | 0.000 | 0.729 | 0.000 | 0.762 | 0.078 | 0.273 | 0.000 | 0.000 | 0.567 | 0.000 |  | 0.027 | 0.000 | 0.000 | 0.131 | 0.000 | 0.000 | 0.000 | 0.000 | 0.000 | 0.002 |
|  | N | 17011 | 17011 | 17011 | 17011 | 17011 | 17011 | 17011 | 17011 | 17011 | 17011 | 17011 | 17011 | 17011 | 17011 | 17011 | 17011 | 17011 | 17011 | 17011 | 17011 | 17011 | 17011 | 17011 | 17011 | 17011 | 17011 |
| Urbanization | Pearson Correlation | 0.009 | 0.011 | .156^**^ | .037^**^ | -0.002 | -.037^**^ | .032^**^ | -.185^**^ | .036^**^ | .019^*^ | .032^**^ | .032^**^ | .042^**^ | .027^**^ | 0.008 | .017^*^ | 1 | .299^**^ | -.226^**^ | .132^**^ | .059^**^ | .239^**^ | -.067^**^ | -.039^**^ | -.063^**^ | .099^**^ |
|  | Sig. (2-tailed) | 0.255 | 0.144 | 0.000 | 0.000 | 0.771 | 0.000 | 0.000 | 0.000 | 0.000 | 0.016 | 0.000 | 0.000 | 0.000 | 0.000 | 0.309 | 0.027 |  | 0.000 | 0.000 | 0.000 | 0.000 | 0.000 | 0.000 | 0.000 | 0.000 | 0.000 |
|  | N | 17011 | 17011 | 17011 | 17011 | 17011 | 17011 | 17011 | 17011 | 17011 | 17011 | 17011 | 17011 | 17011 | 17011 | 17011 | 17011 | 17011 | 17011 | 17011 | 17011 | 17011 | 17011 | 17011 | 17011 | 17011 | 17011 |
| Landform | Pearson Correlation | .017^*^ | .221^**^ | .046^**^ | .027^**^ | .030^**^ | .061^**^ | .039^**^ | -.017^*^ | .052^**^ | 0.001 | .067^**^ | .058^**^ | .048^**^ | 0.011 | .057^**^ | .035^**^ | .299^**^ | 1 | -.137^**^ | -.017^*^ | -.104^**^ | -.049^**^ | .019^*^ | -.221^**^ | -.384^**^ | -.227^**^ |
|  | Sig. (2-tailed) | 0.028 | 0.000 | 0.000 | 0.000 | 0.000 | 0.000 | 0.000 | 0.026 | 0.000 | 0.869 | 0.000 | 0.000 | 0.000 | 0.163 | 0.000 | 0.000 | 0.000 |  | 0.000 | 0.023 | 0.000 | 0.000 | 0.013 | 0.000 | 0.000 | 0.000 |
|  | N | 17011 | 17011 | 17011 | 17011 | 17011 | 17011 | 17011 | 17011 | 17011 | 17011 | 17011 | 17011 | 17011 | 17011 | 17011 | 17011 | 17011 | 17011 | 17011 | 17011 | 17011 | 17011 | 17011 | 17011 | 17011 | 17011 |
| Region | Pearson Correlation | -.064^**^ | .096^**^ | .126^**^ | -0.014 | .110^**^ | .139^**^ | .032^**^ | .020^**^ | 0.005 | 0.000 | 0.007 | .095^**^ | .049^**^ | .029^**^ | .100^**^ | .049^**^ | -.226^**^ | -.137^**^ | 1 | .047^**^ | -.712^**^ | -.566^**^ | -.425^**^ | -.599^**^ | -.447^**^ | -.458^**^ |
|  | Sig. (2-tailed) | 0.000 | 0.000 | 0.000 | 0.075 | 0.000 | 0.000 | 0.000 | 0.008 | 0.475 | 0.952 | 0.376 | 0.000 | 0.000 | 0.000 | 0.000 | 0.000 | 0.000 | 0.000 |  | 0.000 | 0.000 | 0.000 | 0.000 | 0.000 | 0.000 | 0.000 |
|  | N | 17011 | 17011 | 17011 | 17011 | 17011 | 17011 | 17011 | 17011 | 17011 | 17011 | 17011 | 17011 | 17011 | 17011 | 17011 | 17011 | 17011 | 17011 | 17011 | 17011 | 17011 | 17011 | 17011 | 17011 | 17011 | 17011 |
| income | Pearson Correlation | -.089^**^ | -.017^*^ | .245^**^ | .022^**^ | 0.013 | -0.013 | 0.000 | -.170^**^ | 0.005 | .020^**^ | -0.007 | -0.013 | .016^*^ | .018^*^ | -.024^**^ | 0.012 | .132^**^ | -.017^*^ | .047^**^ | 1 | -.090^**^ | -.020^**^ | .026^**^ | -.063^**^ | -.017^*^ | -.039^**^ |
|  | Sig. (2-tailed) | 0.000 | 0.026 | 0.000 | 0.004 | 0.081 | 0.090 | 0.978 | 0.000 | 0.518 | 0.010 | 0.380 | 0.101 | 0.038 | 0.018 | 0.002 | 0.131 | 0.000 | 0.023 | 0.000 |  | 0.000 | 0.010 | 0.001 | 0.000 | 0.024 | 0.000 |
|  | N | 17011 | 17011 | 17011 | 17011 | 17011 | 17011 | 17011 | 17011 | 17011 | 17011 | 17011 | 17011 | 17011 | 17011 | 17011 | 17011 | 17011 | 17011 | 17011 | 17011 | 17011 | 17011 | 17011 | 17011 | 17011 | 17011 |
| Mean Monthly Temperature in January（℃） | Pearson Correlation | .079^**^ | -.158^**^ | -.208^**^ | -0.015 | -.108^**^ | -.140^**^ | -.057^**^ | .182^**^ | -.083^**^ | -.027^**^ | -.017^*^ | -.105^**^ | -.077^**^ | -.034^**^ | -.100^**^ | -.054^**^ | .059^**^ | -.104^**^ | -.712^**^ | -.090^**^ | 1 | .535^**^ | .257^**^ | .668^**^ | .489^**^ | .802^**^ |
|  | Sig. (2-tailed) | 0.000 | 0.000 | 0.000 | 0.055 | 0.000 | 0.000 | 0.000 | 0.000 | 0.000 | 0.000 | 0.024 | 0.000 | 0.000 | 0.000 | 0.000 | 0.000 | 0.000 | 0.000 | 0.000 | 0.000 |  | 0.000 | 0.000 | 0.000 | 0.000 | 0.000 |
|  | N | 17011 | 17011 | 17011 | 17011 | 17011 | 17011 | 17011 | 17011 | 17011 | 17011 | 17011 | 17011 | 17011 | 17011 | 17011 | 17011 | 17011 | 17011 | 17011 | 17011 | 17011 | 17011 | 17011 | 17011 | 17011 | 17011 |
| Mean Monthly rain in January(mm) | Pearson Correlation | .052^**^ | -.079^**^ | -.179^**^ | 0.000 | -.146^**^ | -.212^**^ | -.051^**^ | -.023^**^ | -.038^**^ | .028^**^ | -.033^**^ | -.094^**^ | -.049^**^ | 0.008 | -.093^**^ | -.037^**^ | .239^**^ | -.049^**^ | -.566^**^ | -.020^**^ | .535^**^ | 1 | .626^**^ | .372^**^ | .484^**^ | .567^**^ |
|  | Sig. (2-tailed) | 0.000 | 0.000 | 0.000 | 0.969 | 0.000 | 0.000 | 0.000 | 0.003 | 0.000 | 0.000 | 0.000 | 0.000 | 0.000 | 0.321 | 0.000 | 0.000 | 0.000 | 0.000 | 0.000 | 0.010 | 0.000 |  | 0.000 | 0.000 | 0.000 | 0.000 |
|  | N | 17011 | 17011 | 17011 | 17011 | 17011 | 17011 | 17011 | 17011 | 17011 | 17011 | 17011 | 17011 | 17011 | 17011 | 17011 | 17011 | 17011 | 17011 | 17011 | 17011 | 17011 | 17011 | 17011 | 17011 | 17011 | 17011 |
| Mean Monthly Humidity in January（%） | Pearson Correlation | .036^**^ | -0.010 | -.130^**^ | -0.006 | -.163^**^ | -.202^**^ | -.027^**^ | .031^**^ | 0.004 | .034^**^ | -.031^**^ | -.124^**^ | -.025^**^ | -0.001 | -.124^**^ | -.034^**^ | -.067^**^ | .019^*^ | -.425^**^ | .026^**^ | .257^**^ | .626^**^ | 1 | .451^**^ | .499^**^ | .281^**^ |
|  | Sig. (2-tailed) | 0.000 | 0.197 | 0.000 | 0.451 | 0.000 | 0.000 | 0.000 | 0.000 | 0.608 | 0.000 | 0.000 | 0.000 | 0.001 | 0.941 | 0.000 | 0.000 | 0.000 | 0.013 | 0.000 | 0.001 | 0.000 | 0.000 |  | 0.000 | 0.000 | 0.000 |
|  | N | 17011 | 17011 | 17011 | 17011 | 17011 | 17011 | 17011 | 17011 | 17011 | 17011 | 17011 | 17011 | 17011 | 17011 | 17011 | 17011 | 17011 | 17011 | 17011 | 17011 | 17011 | 17011 | 17011 | 17011 | 17011 | 17011 |
| Mean Monthly rain in July(mm) | Pearson Correlation | .038^**^ | -.191^**^ | -.166^**^ | -.018^*^ | -.144^**^ | -.180^**^ | -.040^**^ | .111^**^ | 0.004 | 0.010 | -.044^**^ | -.135^**^ | -.065^**^ | -.022^**^ | -.144^**^ | -.065^**^ | -.039^**^ | -.221^**^ | -.599^**^ | -.063^**^ | .668^**^ | .372^**^ | .451^**^ | 1 | .819^**^ | .367^**^ |
|  | Sig. (2-tailed) | 0.000 | 0.000 | 0.000 | 0.018 | 0.000 | 0.000 | 0.000 | 0.000 | 0.605 | 0.204 | 0.000 | 0.000 | 0.000 | 0.004 | 0.000 | 0.000 | 0.000 | 0.000 | 0.000 | 0.000 | 0.000 | 0.000 | 0.000 |  | 0.000 | 0.000 |
|  | N | 17011 | 17011 | 17011 | 17011 | 17011 | 17011 | 17011 | 17011 | 17011 | 17011 | 17011 | 17011 | 17011 | 17011 | 17011 | 17011 | 17011 | 17011 | 17011 | 17011 | 17011 | 17011 | 17011 | 17011 | 17011 | 17011 |
| Mean Monthly Humidity in July（%） | Pearson Correlation | 0.002 | -.298^**^ | -.109^**^ | -0.006 | -.133^**^ | -.173^**^ | -.029^**^ | .021^**^ | 0.014 | .035^**^ | -.045^**^ | -.109^**^ | -.052^**^ | -.016^*^ | -.123^**^ | -.065^**^ | -.063^**^ | -.384^**^ | -.447^**^ | -.017^*^ | .489^**^ | .484^**^ | .499^**^ | .819^**^ | 1 | .375^**^ |
|  | Sig. (2-tailed) | 0.796 | 0.000 | 0.000 | 0.435 | 0.000 | 0.000 | 0.000 | 0.006 | 0.063 | 0.000 | 0.000 | 0.000 | 0.000 | 0.038 | 0.000 | 0.000 | 0.000 | 0.000 | 0.000 | 0.024 | 0.000 | 0.000 | 0.000 | 0.000 |  | 0.000 |
|  | N | 17011 | 17011 | 17011 | 17011 | 17011 | 17011 | 17011 | 17011 | 17011 | 17011 | 17011 | 17011 | 17011 | 17011 | 17011 | 17011 | 17011 | 17011 | 17011 | 17011 | 17011 | 17011 | 17011 | 17011 | 17011 | 17011 |
| Mean Monthly Temperature in July（℃） | Pearson Correlation | .065^**^ | -.153^**^ | -.188^**^ | -.022^**^ | -.062^**^ | -.080^**^ | -.054^**^ | .154^**^ | -.104^**^ | -.023^**^ | -.037^**^ | -.070^**^ | -.052^**^ | -.028^**^ | -.051^**^ | -.024^**^ | .099^**^ | -.227^**^ | -.458^**^ | -.039^**^ | .802^**^ | .567^**^ | .281^**^ | .367^**^ | .375^**^ | 1 |
|  | Sig. (2-tailed) | 0.000 | 0.000 | 0.000 | 0.004 | 0.000 | 0.000 | 0.000 | 0.000 | 0.000 | 0.003 | 0.000 | 0.000 | 0.000 | 0.000 | 0.000 | 0.002 | 0.000 | 0.000 | 0.000 | 0.000 | 0.000 | 0.000 | 0.000 | 0.000 | 0.000 |  |
|  | N | 17011 | 17011 | 17011 | 17011 | 17011 | 17011 | 17011 | 17011 | 17011 | 17011 | 17011 | 17011 | 17011 | 17011 | 17011 | 17011 | 17011 | 17011 | 17011 | 17011 | 17011 | 17011 | 17011 | 17011 | 17011 | 17011 |

*. Correlation is significant at the 0.05 level (2-tailed).

**. Correlation is significant at the 0.01 level (2-tailed).
