# Supplementary material for: Prevalence of radiographic knee osteoarthritis in China: a national survey of thirty thousand, four hundred and fifty five individuals cross-sectional study
Source: Int Orthop. 2025 Sep 3;49(10):2489–507. doi: 10.1007/s00264-025-06643-9 (PMC12488765; doi:10.1007/s00264-025-06643-9)
Supplement: Supplementary file 3 — Supplementary Material 3 [file 264_2025_6643_MOESM3_ESM.docx]

Supplementary Table 1 Population-weighted proportion rates of each category of meteorological factors

| **Items** | **Sample size** | **Population-weighted prevalence (per 1,000) and 95% CI** | | |  | **Items** | **Sample size** | **Population-weighted prevalence (per 1,000) and 95% CI** | | | |
| --- | --- | --- | --- | --- | --- | --- | --- | --- | --- | --- | --- |
|  |  | **Male** | **Female** | **Total** |  |  |  | **Male** | **Female** | **Total** |  |
| **Mean monthly temperature** | | |  |  |  | **Mean monthly mount of Relative Humidity** | | |  |  |  |
| January |  |  |  |  |  | January |  |  |  |  |  |
| Q1=-1.54 | 11, 941 | 13.6 (10.9, 16.4) | 27.1 (22.9, 31.3) | 20.2 (16.9, 23.5) |  | Q1=55.8 | 8, 003 | 25.6 (19.8, 31.4) | 43.3 (36.8, 49.8) | 34.5 (28.6, 40.4) |  |
| Q2=0.71 | 3, 552 | 26.8 (21.3, 32.3) | 45.2 (39.4, 50.9) | 36.0 (30.5, 41.4) |  | Q2=69.6 | 7, 731 | 20.8 (16.3, 25.2) | 35.3 (30.3, 40.3) | 28.2 (23.8, 32.5) |  |
| Q3=4.35 | 7, 534 | 15.8 (15.0, 16.6) | 32.0 (28.7, 35.3) | 24.1 (22.1, 26.1) |  | Q3=76.13 | 9, 664 | 13.4 (10.5, 16.3) | 28.1 (22.3, 33.9) | 20.4 (16.1, 24.8) |  |
| Q4=20.21 | 7, 428 | 22.0 (17.7, 26.2) | 36.8 (31.7, 41.9) | 29.4 (25.2, 33.6) |  | Q4=82.93 | 5, 057 | 17.1 (15.3, 18.9) | 32.8 (29.4, 36.2) | 25.0 (22.6, 27.4) |  |
| P-value for trend test |  | 0.349 | 0.502 | 0.349 |  | P-value for trend test |  | 0.001 | 0.004 | 0.001 |  |
| July |  |  |  |  |  | July |  |  |  |  |  |
| Q1=27.4 | 11, 929 | 13.7 (11.0, 16.5) | 27.0 (22.7, 31.2) | 20.1 (16.8, 23.5) |  | Q1=70.6 | 11, 998 | 17.6 (14.7, 20.5) | 32.8 (28.3, 37.3) | 25.2 (21.7, 28.7) |  |
| Q2=28.44 | 9, 645 | 27.6 (22.3, 32.9) | 46.2 (40.8, 51.6) | 36.9 (31.8, 42.0) |  | Q2=77.07 | 4, 041 | 27.1 (20.9, 33.3) | 46.2 (40.1, 52.3) | 36.7 (30.7, 42.6) |  |
| Q3=28.57 | 3, 519 | 15.5 (14.9, 16.0) | 31.5 (28.5, 34.5) | 23.7 (21.9, 25.4) |  | Q3=80.40 | 10, 399 | 19.1 (14.7, 23.6) | 33.5 (28.5, 38.5) | 26.4 (21.9, 30.8) |  |
| Q4=29.46 | 5, 362 | 21.5 (17.6, 25.4) | 36.6 (32.1, 41.1) | 29.1 (25.3, 32.8) |  | Q4=84.73 | 4, 017 | 16.7 (15.1, 18.3) | 32.0 (29.1, 34.8) | 24.3 (22.4, 26.3) |  |
| P-value for trend test |  | 0.402 | 0.530 | 0.408 |  | P-value for trend test |  | 0.080 | 0.056 | 0.054 |  |
| **Mean monthly amount of Rainfall** | | |  |  |  |  |  |  |  |  |  |
| January |  |  |  |  |  |  |  |  |  |  |  |
| Q1=2.69 | 7, 778 | 25.2 (19.1, 31.3) | 42.7 (35.7, 49.7) | 33.9 (27.6, 40.3) |  |  |  |  |  |  |  |
| Q2=7.31 | 7, 535 | 14.3 (11.6, 17.1) | 28.3 (24.2, 32.4) | 21.1 (17.6, 24.5) |  |  |  |  |  |  |  |
| Q3=35.77 | 8, 194 | 21.9 (17.9, 25.9) | 37.4 (32.6, 42.2) | 29.7 (25.8, 33.7) |  |  |  |  |  |  |  |
| Q4=72.06 | 6, 948 | 15.8 (14.9, 16.7) | 31.4 (28.6, 34.2) | 23.8 (22.1, 25.5) |  |  |  |  |  |  |  |
| P-value for trend test |  | 0.067 | 0.064 | 0.066 |  |  |  |  |  |  |  |
| July |  |  |  |  |  |  |  |  |  |  |  |
| Q1=130.55 | 9, 033 | 16.1 (12.8, 19.4) | 30.7 (25.8, 35.7) | 23.3 (19.3, 27.3) |  |  |  |  |  |  |  |
| Q2=138.78 | 6, 314 | 26.3 (19.6, 32.9) | 45.0 (38.0, 52.0) | 35.6 (28.9, 42.2) |  |  |  |  |  |  |  |
| Q3=211.63 | 9, 938 | 15.1 (13.6, 16.6) | 29.7 (27.4, 32.1) | 22.5 (20.5, 24.5) |  |  |  |  |  |  |  |
| Q4=284.14 | 5, 170 | 21.6 (17.5, 25.7) | 36.9 (32.1, 41.8) | 29.4 (25.4, 33.4) |  |  |  |  |  |  |  |
| P-value for trend test |  | 0.756 | 0.972 | 0.856 |  |  |  |  |  |  |  |

*The trend test was performed using the Cochran-Armitage test to assess the trend of prevalence changes with stratifying factors.

Supplementary Table 2 Single-factor analysis of the male modeling population

| **Items** |  | **Total** | **Knee arthritis** | | | ***P*** |
| --- | --- | --- | --- | --- | --- | --- |
|  |  |  | **Yes** | **No** | |  |
| **Age（years）** | 50-59 | 3686(31.4) | 466(18.5) | 3220(34.9) | | ＜0.001 |
|  | 60-69 | 4768(40.6) | 1014(40.2) | 3754(40.7) | |  |
|  | 70-79 | 2553(21.7) | 763(30.2) | 1790(19.4) | |  |
|  | ≥80 | 746(6.4) | 282(11.2) | 464(5.0) | |  |
| **Ethnicity** | Han | 11426(97.2) | 2457(97.3) | 8969(97.2) | | 0.758 |
|  | Other | 327(2.8) | 68(2.7) | 259(2.8) | |  |
| **Education** | Illiterate | 1225(10.4) | 412(16.3) | 813(8.8) | | ＜0.001 |
|  | Primary school | 5211(44.3) | 1240(49.1) | 3971(43.0) | |  |
|  | Junior high school | 3552(30.2) | 616(24.4) | 2936(31.8) | |  |
|  | Senior high school | 1308(11.1) | 197(7.8) | 1111(12.0) | |  |
|  | College or above | 457(3.9) | 60(2.4) | 397(4.3) | |  |
| **Born in famine year** | No | 10993(93.5) | 2415(95.6) | 8578(93.0) | | ＜0.001 |
|  | Yes | 760(6.5) | 110(4.4) | 650(7.0) | |  |
| **BMI（kg/m^2^）** | ＜18.5 | 268(2.3) | 50(2.0) | 218(2.4) | | ＜0.001 |
|  | 18.5-23.9 | 6416(54.56) | 1282(50.8) | 5134(55.6) | |  |
|  | 24-27.9 | 4030(34.3) | 917(36.3) | 3113(33.7) | |  |
|  | ≥28 | 1039(8.8) | 276(10.9) | 763(8.3) | |  |
| **Central Obesity** | No | 8408(71.5) | 1681(66.6) | 6727(72.9) | | ＜0.001 |
|  | Yes | 3345(28.45) | 844(33.4) | 2501(27.1) | |  |
| **Previous Knee Injury** | No | 11668(99.3) | 2502(99.1) | 9166(99.3) | | 0.209 |
|  | Yes | 85(0.7) | 23(0.9) | 62(0.7) |  |  |
| **Cigarette smoking** | No | 7573(64.4) | 1802(71.4) | 5771(62.5) | | ＜0.001 |
|  | Yes | 4180(35.6) | 723(28.6) | 3457(37.5) | |  |
| **Alcohol consumption** | No | 7892(67.2) | 1843(73.0) | 6049(65.6) | | ＜0.001 |
|  | Yes | 3861(32.9) | 682(27.0) | 3179(34.5) | |  |
| **VD** | No | 11647(99.1) | 2500(99.0) | 9147(99.1) | | 0.597 |
|  | Yes | 106(0.9) | 25(1.0) | 81(0.9) |  |  |
| **Hypertension** | No | 9101(77.4) | 1815(71.9) | 7286(79.0) | | ＜0.001 |
|  | Yes | 2652(22.6) | 710(28.1) | 1942(21.0) | |  |
| **Diabetes** | No | 11079(94.3) | 2394(94.8) | 8685(94.1) | | 0.182 |
|  | Yes | 674(5.7) | 131(5.2) | 543(5.9) | |  |
| **Digestive drug** | No | 11504(97.9) | 2472(97.9) | 9032(97.9) | | 0.938 |
|  | Yes | 249(2.1) | 53(2.1) | 196(2.1) | |  |
| **Anti-hypertension drug** | No | 9681(82.4) | 1947(77.1) | 7734(83.8) | | ＜0.001 |
|  | Yes | 2072(17.6) | 578(22.9) | 1494(16.2) | |  |
| **Anti-glucose drug** | No | 11204(95.3) | 2410(95.4、5) | 8794(95.3) | | 0.754 |
|  | Yes | 549(4.7) | 115(4.6) | 434(4.7) | |  |
| **Urbanization** | Rural area | 7209(61.3) | 1666(66.0) | 5543(60.1) | | ＜0.001 |
|  | Urban area | 4544(38.7) | 859(34.0) | 3685(40.0) | |  |
| **Region** | Innerland | 8064(68.6) | 1727(68.4) | 6337(68.67) | | 0.792 |
|  | Coastal | 3689(31.4) | 798(31.6) | 2891(31.3) | |  |
| **Landform** | Plain | 8930(76.0) | 1928(76.4) | 7002(75.9) | | ＜0.001 |
|  | Mountainous area | 65(0.6) | 18(0.7) | 47(0.5) | |  |
|  | Plateau | 941(8.0) | 153(6.1) | 788(8.5) | |  |
|  | Hill | 1391(11.8) | 310(12.3) | 1081(11.7) | |  |
|  | Basin | 426(3.6) | 116(4.6) | 310(3.4) | |  |
| **Mean Monthly income (RMB/month)** | ＜1000 | 1050(8.9) | 275(10.9) | 775(8.4) | | ＜0.001 |
|  | 1000-1999 | 9372(79.7) | 2015(79.8) | 7357(79.7) | |  |
|  | ≥2000 | 1331(11.3) | 235(9.3) | 1096(11.9) | |  |
| **Mean Monthly Temperature in January（℃）** | | 0.7(5.9) | 1.8(12.89) | 0.7(3.1) | | 0.053 |
| **Mean Monthly Temperature in July（℃）** | | 27.6(1.2) | 28.0(3.0) | 27.5(0.1) | | 0.152 |
| **Mean Monthly rain in January(mm)** | | 7.3(32.6) | 13.5(36.5) | 7.3(0.1) | | ＜0.001 |
| **Mean Monthly rain in July(mm)** | | 138.8(81.1) | 164.9(79.5) | 93.0(71.2) | | 0.113 |
| **Mean Monthly Humidity in January（%）** | | 69.6(20.3) | 71.5(20.3) | 61.5(6.8) | | ＜0.001 |
| **Mean Monthly Humidity in July（%）** | | 77.1(9.8) | 77.7(9.8) | 66.4(9.0) | | ＜0.001 |

Supplementary Table 3 Single-factor analysis of the female modeling population

| **Items** |  | **Total** | **Knee arthritis** | | | ***P*** |
| --- | --- | --- | --- | --- | --- | --- |
|  |  |  | **Yes** | **No** | |  |
| **Age（years）** | 50-59 | 5285(35.2) | 1179(21.0) | 4106(43.6) | | ＜0.001 |
|  | 60-69 | 6008(40.0) | 2343(41.7) | 3665(38.9) | |  |
|  | 70-79 | 2957(19.7) | 1617(28.8) | 1340(14.2) | |  |
|  | ≥80 | 784(5.2) | 481(8.6) | 303(3.2) | |  |
| **Ethnicity** | Han | 14621(97.3) | 5466(97.3) | 9155(97.2) | | 0.968 |
|  | Other | 413(2.7) | 154(2.7) | 259(2.8) | |  |
| **Education** | Illiterate | 2497(16.6) | 1199(21.3) | 1298(13.8) | | ＜0.001 |
|  | Primary school | 7005(46.6) | 2821(50.2) | 4184(44.4) | |  |
|  | Junior high school | 3742(24.9) | 1118(19.9) | 2624(27.9) | |  |
|  | Senior high school | 1459(9.7) | 402(7.2) | 1057(11.2) | |  |
|  | College or above | 331(2.2) | 80(1.4) | 251(2.7) | |  |
| **Born in famine year** | No | 13956(92.8) | 5377(95.7) | 8579(91.1) | | ＜0.001 |
|  | Yes | 1078(7.2) | 243(4.3) | 835(8.9) | |  |
| **BMI（kg/m^2^）** | ＜18.5 | 521(3.5) | 189(3.4) | 332(3.5) | | ＜0.001 |
|  | 18.5-23.9 | 7278(48.4) | 2380(42.3) | 4898(52.0) | |  |
|  | 24-27.9 | 5388(35.8) | 2129(37.9) | 3259(34.6) | |  |
|  | ≥28 | 1847(12.3) | 922(16.4) | 925(9.8) | |  |
| **Central Obesity** | No | 10558(70.2) | 3531(62.8) | 7027(74.6) | | ＜0.001 |
|  | Yes | 4476(29.8) | 2089(37.2) | 2387(25.4) | |  |
| **Previous Knee Injury** | No | 14899(99.1) | 5554(98.8) | 9345(99.3) | | 0.006 |
|  | Yes | 135(0.9) | 66(1.2) | 69(0.7) |  |  |
| **Children** | ≤1 | 3599(23.9) | 861(15.3) | 2738(29.1) | | ＜0.001 |
|  | 2 | 5514(36.7) | 1952(34.7) | 3562(37.8) | |  |
|  | ≥3 | 5921(39.4) | 2807(49.9) | 3114(33.1) | |  |
| **Cigarette smoking** | No | 14770(98.2) | 5552(98.8) | 9218(97.9) | | ＜0.001 |
|  | Yes | 264(1.8) | 68(1.2) | 196(2.1) | |  |
| **Alcohol consumption** | No | 14862(98.9) | 5569(99.1) | 9293(98.7) | | 0.035 |
|  | Yes | 172(1.1) | 51(0.9) | 121(1.3) | |  |
| **VD** | No | 14773(98.3) | 5526(98.3) | 9247(98.2) | | 0.645 |
|  | Yes | 261(1.7) | 94(1.7) | 167(1.8) |  |  |
| **Hypertension** | No | 11336(75.4) | 3905(69.5) | 7431(78.9) | | ＜0.001 |
|  | Yes | 3698(24.6) | 1715(30.5) | 1983(21.1) | |  |
| **Diabetes** | No | 14068(93.6) | 5181(92.2) | 8887(94.4) | | ＜0.001 |
|  | Yes | 966(6.4) | 439(7.8) | 527(5.6) | |  |
| **Digestive drug** | No | 14673(97.6) | 5456(97.1) | 9217(97.9) | | 0.001 |
|  | Yes | 361(2.4) | 164(2.9) | 197(2.1) | |  |
| **Anti-hypertension drug** | No | 11961(79.6) | 4165(74.1) | 7796(82.8) | | ＜0.001 |
|  | Yes | 3073(20.4) | 1455(25.9) | 1618(17.2) | |  |
| **Anti-glucose drug** | No | 14242(94.7) | 5241(93.3) | 9001(95.6) | | ＜0.001 |
|  | Yes | 792(5.3) | 379(6.7) | 413(4.4) | |  |
| **Urbanization** | Rural area | 9201(61.2) | 3560(63.3) | 5641(59.9) | | ＜0.001 |
|  | Urban area | 5833(38.8) | 2060(36.7) | 3773(40.1) | |  |
| **Region** | Innerland | 4599(30.6) | 1705(30.3) | 2894(30.7) | | 0.604 |
|  | Coastal | 10435(69.4) | 3915(69.7) | 6520(69.3) | |  |
| **Landform** | Plain | 11410(75.9) | 4262(75.8) | 7148(75.9) | | ＜0.001 |
|  | Mountainous area | 84(0.6) | 40(0.7) | 44(0.5) | |  |
|  | Plateau | 1287(8.6) | 446(7.9) | 841(8.9) | |  |
|  | Hill | 1720(11.4) | 606(10.8) | 1114(11.8) | |  |
|  | Basin | 533(3.5) | 266(4.7) | 267(2.8) | |  |
| **Mean Monthly income (RMB/month)** | ＜1000 | 1594(10.6) | 647(11.5) | 947(10.1) | | 0.007 |
|  | 1000-1999 | 12101(80.5) | 4502(80.1) | 7599(80.7) | |  |
|  | ≥2000 | 1339(8.9) | 471(8.4) | 868(9.2) | |  |
| **Mean Monthly Temperature in January（℃）** | | 1.78(11.9) | -1.37(6.4) | 1.78(14.6) | | 0.040 |
| **Mean Monthly Temperature in July（℃）** | | 28.04(1.2) | 27.53(1.2) | 28.04(3.0) | | 0.347 |
| **Mean Monthly rain in January(mm)** | | 13.51(36.5) | 11.59(33.5) | 13.51(36.3) | | ＜0.001 |
| **Mean Monthly rain in July(mm)** | | 164.88(79.5) | 138.78(79.5) | 164.88(79.5) | | 0.002 |
| **Mean Monthly Humidity in January（%）** | | 70.6(20.3) | 69.6(20.3) | 71.53(20.3) | | ＜0.001 |
| **Mean Monthly Humidity in July（%）** | | 77.67(9.8) | 75.33(9.8) | 77.67(9.8) | | ＜0.001 |
